# Supplementary material for: Phenome-wide association studies across large population cohorts support drug target validation
Source: Nat Commun. 2018 Oct 16;9:4285. doi: 10.1038/s41467-018-06540-3 (PMC6191429; doi:10.1038/s41467-018-06540-3)
Supplement: Supplementary file 1 — Supplementary Information [file 41467_2018_6540_MOESM1_ESM.pdf]

Phenome-wide association studies across large population cohorts support drug target validation

Diogo et al.

SUPPLEMENTARY INFORMATION

Supplementary Methods **3**
Gene selection **3**

Supplementary Notes **9**
Supplementary Note 1. List of published studies in the Genomics plc GWAS database used in this study 9

Supplementary Figures **11**
Supplementary Fig. 1. Replication of known GWAS associations through meta-analysis of PheWAS results from four disease-agnostic cohorts with extensive health information 11
Supplementary Fig. 2. Effect sizes of known GWAS associations in the meta-PheWAS 12
Supplementary Fig. 3. Effect sizes of known GWAS associations in the disease-agnostic cohorts PheWAS 13
Supplementary Fig. 4. Effects of replicated and putative novel associations (FDR<0.1). 14
Supplementary Fig. 5. Effect sizes of putative novel associations observed in the individual cohorts and in the meta-analysis. 17
Supplementary Fig. 6. Independent associations for galectin-3 plasma levels and Parkinson's disease risk at the *GCH1-LGALS3* locus 18
Supplementary Fig. 7. Phenotype-phenotype correlations in 23andMe. 19
Supplementary Fig. 8. Association of *PNPLA3* rs738409-G with multiple disease-relevant endpoints. 20

Supplementary Tables **21**
Supplementary Table 1. SNP-phenotype associations reported earlier in published GWAS for the 25 SNPs interrogated through PheWAS in this study 21
Supplementary Table 2. Examples of mapped phenotypic endpoints 23
Supplementary Table 3. Mapped phenotypes found in at least two disease-agnostic cohorts and included in the meta-analysis 24
Supplementary Table 4. Replication of known GWAS associations in the PheWAS meta-analysis 29
Supplementary Table 5. Putative novel associations identified in the meta-PheWAS 32
Supplementary Table 6. Replication of potential novel associations in the latest release of UK Biobank 35
Supplementary Table 7. Novel association of the *PNPLA3* SNP rs738409 in the 23andMe cohort, before and after adjusting for elevated liver test 37
Supplementary Table 8. Association of the *IFIH1* SNP rs2111485 (in complete LD with the *IFIH1* missense SNP rs1990760) with asthma in 23andMe cohort, before and after adjusting for autoimmune diseases 38

Supplementary References **39**

Supplementary Methods

Gene selection

A short description of the selected candidate target genes and level of genetic and/or biological support for a link to common human disease is provided. All SNPs selected for PheWAS in this study (bold) had reported genome-wide significant GWAS associations (P<5e-8) in the European population.

A. Autoimmunity	
ATG16L1	*ATG16L1* encodes the Autophagy-related protein 16 like 1. SNPs at the *ATG16L1* locus are unequivocally associated with Crohn's disease (CD) and inflammatory bowel disease (IBD)[1-3]. The missense SNP rs2241880 (p.T300A) has been proposed as a causal risk SNP. pT300A renders the ATG16L1 protein susceptible to degradation by caspase-3, which leads to reduced autophagy, elevated inflammatory cytokine response, and reduced pathogen clearance[4, 5]. The intronic variant **rs13391356** analyzed here is in tight linkage disequilibrium (LD) (r^2=0.99) with rs2241880. It is hypothesized that stabilizing ATG16L1 protein and/or enhancing its function may be of therapeutic benefit to CD and IBD patients.
CARD9	*CARD9* encodes the recruitment domain-containing protein 9. Independent SNPs at the *CARD9* locus, including the missense SNP **rs4077515** (p.S12N) and the intronic SNP **rs11145766**, are consistently associated with an increased risk of IBD, CD and ulcerative colitis (UC) in GWAS[6, 7]. The IBD risk allele rs4077515-T increases *CARD9* mRNA levels in blood[8]. Conversely, the splice-site variant IVS11+1G>C creates a premature termination codon that causes loss-of-function and is associated with protection from IBD[9]. Complete loss of CARD9 leads to susceptibility to fungal infections[10], consistent with its biological function as a molecular scaffold for the assembly of a BCL10 signaling complex that activates NF-kappaB[11]. It is hypothesized that moderate reduction of CARD9 function may be of therapeutic benefit to CD and IBD patients.
CD226	CD226, also called DNAX accessory molecule (DNAM-1), is an activating immunoreceptor expressed at the surface of natural killer (NK) cells, T cells, monocytes and platelets. The gene has been described to play an important role in NK cells function[12, 13]. SNPs at the *CD226* locus, including the missense SNP **rs763361** (p.S307G), are associated with IBD, T1D and mean platelet volume (MPV) in the European population, with the allele increasing IBD and T1D risk being associated with decreased MPV[3, 14, 15]. Studies suggested a decreased expression of CD226 in patients with autoimmune diseases, consistent with the rs763361 autoimmunity risk allele being associated with reduced expression of CD226[16, 17].
GPR35	The G protein-coupled receptor 35 (GPR35) is a recently deorphanized GPCR detected in numerous tissues, including the gastrointestinal tract, the immune system, and the cardiovascular system[18]. SNPs at the *GPR35* locus, including the *GPR35* missense SNP **rs3749171** (p.T139M), have been found associated with UC and IBD[3].

GPR65	*GPR65* encodes the G protein coupled receptor 65, also known as T-cell death associated gene 8 (TDAG8). GWAS have identified SNPs at the *GPR65* locus as associated with CD and UC[3]. The lead SNP, rs8005161, is in prefect LD with the *GPR65* missense SNP **rs3742704** (p.I231L). In functional studies, I231L was shown to alter GPR65 signaling and to affect lysosomal pH, leading to lysosomal dysfunction and impaired bacterial restriction[19].
IFIH1	*IFIH1* encodes the Interferon-induced helicase C domain-containing protein 1, also known as Melanoma differentiation-associated protein 5 (MDA5), a cytoplasmic sensor of viral nucleic acids that plays a major role in the activation of a cascade of antiviral responses including the induction of type I interferons and pro-inflammatory cytokines[20]. Rare gain-of-function (GOF) mutations in *IFIH1* have been reported to cause Aicardi-Goutières syndrome and Singleton-Merten syndrome 1, two syndromes characterized by high interferon signaling[21-23]. Conversely, rare loss-of-function (LOF) variants that lead to severe disruption of *IFIH1* signaling function have been recently shown to cause a primary immunodeficiency manifested in extreme susceptibility to common respiratory RNA viruses [24, 25]. In addition, an allelic series of rare, low-frequency and common LOF alleles (rs35744605, **rs35667974**, **rs1990760**) has been shown to protect against multiple autoimmune diseases, including type 1 diabetes (T1D), vitiligo, lupus and psoriasis, while rs1990760 and rs35667974 were found associated with increased risk of ulcerative colitis [26-36]. The rare T1D protective rs35744605 (p.E627X) and rs35667974 (p.I923V) alleles confer a significant inhibition of IFNβ production, and the common T1D protective allele rs1990760 (p.T946A) has been shown to reduce MDA5 expression following IFNβ treatment[27, 32]. Furthermore, mice heterozygote or homozygote for the rs1990760 T1D risk allele exhibit higher interferon signature, enhanced resistance to viral challenge, and higher incidence of streptozocin-induced T1D [37]. Based on the genetic findings, it is hypothesized that MDA5 inhibition might be of therapeutic benefit for patients diagnosed with several autoimmune diseases.
IRF5	The interferon regulatory factor 5 (IRF5) is involved in the activation of type I interferon signaling in response to viral infection. Common GWAS SNPs are associated with multiple autoimmune diseases. Two independent signals at the locus, driven by **rs10488631** and **rs2004640**, are associated with rheumatoid arthritis (RA), primary biliary cirrhosis (PBC), systemic lupus erythematosus (SLE), and Sjogren's syndrome[35, 38-40]. In addition, a SNP in LD with rs10488631 (rs62478615) was reported associated with systemic sclerosis[41], and **rs3823536** (in LD with rs2004640) has been reported associated with ulcerative colitis[3]. Multiple studies evaluating the function of rs2004640 have shown the autoimmune risk allele to increase IRF5 mRNA and protein levels in immune cells, upregulate IFNα-induced genes, and increase pro-inflammatory cytokine production by monocyte-derived dendritic cells and monocyte-derived macrophages[42-44]. Based on the genetic findings, it is hypothesized that IRF5 inhibition might be of therapeutic benefit for patients diagnosed with autoimmune diseases.

TYK2	The Tyrosine kinase 2 (TYK2) is a member of the Janus kinase (JAK) family of proteins that mediates signaling downstream of several cytokine receptors. TYK2-signaling deficient patients carrying rare *TYK2* LOF mutations and characterized by immunodeficiency have been reported[45, 46]. In GWAS, independent low-frequency (**rs34536443**) and common (**rs12720356**) *TYK2* missense SNPs have been associated with a variety of autoimmune diseases, including RA, SLE, IBD, Psoriasis, and multiple sclerosis (MS)[3, 35, 38, 47-49]. The protective alleles have been reported to decrease TYK2 kinase activity and downstream signaling in immune cells[50, 51]. It is hypothesized that TYK2 inhibition might be of therapeutic benefit for patients diagnosed with autoimmune diseases.
B. Immunity	
CDHR3	The cadherin-related family, member 3 (CHDR3) transmembrane protein is involved in cell adhesion, epithelial polarity, cell-cell interaction and differentiation, and is predominantly expressed in respiratory epithelium. A GWAS in Danish asthma patients identified the coding SNP **rs6967330** (p.C529Y) in *CDHR3* as associated with recurrent childhood asthma exacerbation[52]. The disease-associated SNP locates to the protein's extracellular domain and is associated with increased CDHR3 cell surface expression. This SNP was shown to stimulate cell internalization of rhinovirus C, a known stimulus for asthma exacerbation, in cultured cells[53]. It is hypothesized that inhibition of CDHR3 might reduce rhinovirus C internalization and virus-induced asthma-related symptoms.
C. Cardiovascular & metabolic diseases	
F11	Factor XI is a serine protease within the contact pathway of the coagulation cascade. In humans, Factor XI is encoded by *F11*. Deficiency of factor XI causes the rare condition hemophilia C which affects up to 8% of the Ashkenazi Jewish population[54]. Homozygotes and compound-heterozygotes for *F11* LOF mutations show little spontaneous bleeding, but surgical procedures may cause excessive blood loss, requiring prophylaxis. High levels of factor XI have been proposed to increase the risk of thrombosis. Common SNPs near *F11*, including rs4253417, rs1593, and **rs4253399**, have been associated with altered Factor XI plasma levels, activated partial thromboplastin time (aPTT) and venous thromboembolism risk[55-57]. FXI-reducing drugs are under development for the prevention of venous thrombosis[58].
F12	Coagulation factor XII, (Hageman factor) is a plasma serine protease and a zymogen form of factor XIIa, which is encoded by the *F12* gene. Factor XII is part of the coagulation cascade and activates factor XI and prekallikrein *in vitro*. Factor XII itself is activated by negatively charged surfaces and is the starting point of the intrinsic pathway. Factor XII deficiency is rare and inherited in an autosomal recessive manner[59, 60]. Typically, Factor XII deficiency is asymptomatic and, unlike FXI deficiency, does not result in excess bleeding. A suggested GOF mutation in *F12* has been reported in patients with hereditary angioedema [61]. Knockout mice are less susceptible to thrombosis[62]. Factor XII appears to be involved also in the later stages of clot formation. Common variants in *F12*

	(rs2545801, **rs2731672**) have been associated with Factor XII levels, aPTT and plasma levels of various vasoactive substances including endothelin, bradykinin, adrenomedullin, chromogranin, and phenylalanine[63, 64]. Inhibition of Factor XII has been considered a therapeutic strategy for the prevention of thromboembolism.
GDF15	Growth differentiation factor-(GDF-)15 is a stress-responsive cytokine emerging as a biomarker of cardiac and vascular dysfunction and disease. Elevated circulating levels of GDF-15 identify high-risk individuals across the cardiovascular continuum, from stable coronary artery disease to acute coronary syndrome and heart failure. The association of GDF-15 with outcome in these conditions is independent of clinical risk factors and established biomarkers, including NT-proBNP (N-terminal pro-B-type natriuretic peptide) and troponin. Common SNPs associated with BMI by the GIANT consortium have been identified in the proximal region of the *GDF15* gene[65]. The lead SNP **rs17724992** is an intronic SNP in *PGPEP1* where the minor allele associated with decreased BMI. *GDF15* was annotated as a gene with biological relevance to this locus in the original publication, but eQTL data does not support a direct functional effect on *GDF15* mRNA levels [66]. Increased serum levels of GDF-15 have been reported associated with type 2 diabetes (T2D) and impaired fasting glucose[67].
GUCY1A3	Soluble guanylate cyclase (sGC) is an intracellular enzyme that mediates the nitric oxide (NO) signaling pathway. It catalyzes synthesis of the second messenger cyclic guanosine monophosphate (cGMP), which leads to vasorelaxation and inhibits smooth muscle proliferation, leukocyte recruitment and platelet aggregation through a number of downstream mechanisms. In humans, sGC subunits are encoded by the genes *GUCY1A2*, *GUCY1A3*, *GUCY1B2* and *GUCY1B3*. An sGC activator, Ricoguat, is a marketed therapeutic in cardiopulmonary disease. Common SNPs near *GUCY1A3* (**rs13139571**) have been significantly associated with diastolic blood pressure, and suggestively associated with systolic blood pressure and hypertension[68]. The same SNPs are also associated with coronary artery disease and myocardial infarction[68]. rs13139571 is an intronic SNP in *GUCY1A3* and an eQTL for *GUCY1A3* in multiple tissues. Knockout of *GUCY1A3* or *GUCY1B3* in murine models results in hypertension[69]. Truncating mutations have been observed in patients with moyamoya disease with achalasia[70].
KNG1	Kininogen-1, encoded by the KNG1 gene, is the precursor protein to high-molecular-weight kininogen (HMWK), low-molecular-weight kininogen (LMWK), and bradykinin. Alternative splicing of the *KNG1* gene leads to either low-molecular-weight kininogen (LMWK) or high-molecular-weight kininogen (HMWK) which can be cleaved to produce bradykinin. HMWK is essential for blood coagulation and assembly of the kallikrein-kinin system. HMWK interacts with Factor XII, Factor XI and prekallikrein where it acts as a cofactor to initiate the contact activation (intrinsic) pathway of coagulation. HMWK is not enzymatically active, but functions as a cofactor for the activation of kallikrein, factor XII and activation of factor XI by factor XIIa. GWAS have identified SNPs at the *KNG1* locus (**rs5030062** and rs710446, both in strong LD) that associate with des-arg(9) bradykinnin levels, Factor XI plasma levels and aPTT[55-57].

LGALS3	Galectin-3 (Gal-3) is a multifunctional protein of an expanding family of β-galactoside–binding animal lectins, mainly produced by macrophages and monocytes. It is implicated in a variety of biologic events, such as inflammation, angiogenesis and fibrotic disorders. Gal-3 is broadly expressed in various tissues and secreted into the extracellular matrix and circulation. Increased circulating levels of galectin-3 have been associated with various diseases, including cancer, immunological disorders, and heart failure. A GWAS of plasma galectin-3 levels identified **rs2274273** (a non-coding variant in LD with missense variants in *LGALS3*) as associated with reduced levels of circulating galectin-3[71]. No specific disease associations have been reported for this variant.
PNPLA3	Patatin-like phospholipase domain-containing protein 3 (PNPLA3) also known as adiponutrin, acylglycerol O-acyltransferase or calcium-independent phospholipase A2-epsilon (iPLA2-epsilon), is an enzyme primarily expressed in the liver and adipose which partitions between membranes and lipid droplets. PNPLA3 hydrolyzes acylglycerols with maximal activity against triacylglycerol, diacylglycerol, and monoacylglycerol. A polymorphism (**rs738409**, p.I148M) in *PNPLA3* has been associated with Alcoholic and Non-alcoholic Fatty Liver Disease (NAFLD), Non-alcoholic Steatohepatitis (NASH), Liver Cirrhosis, Hepatic Cell Carcinoma, and Type 2 diabetes, although the mechanistic basis for these associations is not entirely resolved[72-76]. *Pnlpa3* knockout mice failed to demonstrate significant increases in liver fat. Overexpressing human PNPLA3 in mouse liver did not increase steatosis, but overexpression of the human p.I148M variant did recapitulate the fatty liver phenotype in mice, and metabolic studies suggested a complex mechanism[75]. Knock-in of the human p.I148M variant at endogenous levels showed that accumulation of triacylglycerides in the liver is associated with an enrichment of catalytically inactive PNPLA3 on lipid droplets[76].
SLC30A8	Solute carrier family 30, member 8 (SLC30A8, or Znt8) is a zinc transporter highly expressed in the pancreas and implicated in insulin secretion in humans. Early GWAS studies by Sladek et al.[77] associated the missense allele Arg325Trp (**rs13266634**) with susceptibility to T2D. The T2D risk allele was shown to increase fasting proinsulin, fasting insulin and fasting glucose, and was found associated with lower insulinogenic index and HOMA derived β-cell function, suggesting a defect in β-cell insulin processing, secretion or insulin clearance. Humans homozygous for the diabetes risk variant exhibited a post–oral glucose increase in the C-peptide:insulin ratio. Functional studies of the p.Arg325Trp risk variant have reported conflicting effects on zinc transporter activity. However, in 2014, large scale sequencing of *SLC30A8* in multiple populations demonstrated that heterozygosity for truncating LOF mutations in *SLC30A8* results in substantial protection from the risk of T2D[78].
D. Neurodegeneration	
LRRK2	Mutations in *LRRK2* are the most common genetic cause of both familial and sporadic Parkinson's disease (PD). The most common PD mutation, p.G2019S (rs34637584), located within the LRRK2 kinase domain, causes autosomal dominant PD. G2019S increases kinase activity, auto-phosphorylation and

	phosphorylation of generic substrates, and is enriched in Ashkenazi Jews and North African Arab Berbers[79]. Non-coding variants at the *LRRK2* locus (e.g., **rs1491942**) are associated with increased *LRRK2* expression in multiple cell types and increased risk of sporadic PD through GWAS[80]. Together, the genetic and functional data suggest that inhibition of LRRK2's kinase function is a potential therapeutic mechanism for the treatment of PD patients with the G2019S mutation, and perhaps more broadly patients with PD due to other etiologies. Both, p.G2019S and another coding variant, **rs11564187** (p.N2081D), are also unequivocally associated with risk of CD and UC[3, 9].
TMEM175	GWAS and conditional analyses identified **rs34311866** (p.M393T) and **rs34884217** (p.Q65P) within *TMEM175* at the *GAK*/*TMEM175*/*DGKQ* locus as associated with the risk of PD[80]. TMEM175 is a lysosomal potassium channel that regulates lysosomal K(+) permeability[81]. Deficiency for TMEM175 in neuronal cells impairs lysosomal function and increases α-synuclein aggregation[82]. Modification of TMEM175's K(+) channel activity is considered a potential therapeutic mechanism for the treatment of PD patients.

Supplementary Notes

Supplementary Note 1. List of published studies in the Genomics plc GWAS database used in this study

Berndt, SI. et al. Genome-wide meta-analysis identifies 11 new loci for anthropometric traits and provides insights into genetic architecture. *Nat Genet.* **45**, 501-12. doi: 10.1038/ng.2606. (2013).
Bradfield, JP. et al. A genome-wide association meta-analysis identifies new childhood obesity loci. *Nat Genet.* **44**, 526-31. doi: 10.1038/ng.2247. (2012). (www.egg-consortium.org)
Buch, S. et al. A genome-wide association study confirms PNPLA3 and identifies TM6SF2 and MBOAT7 as risk loci for alcohol-related cirrhosis. *Nat Genet.* **47**, 1443-8. doi: 10.1038/ng.3417. (2015).
CONVERGE consortium et al. Sparse whole-genome sequencing identifies two loci for major depressive disorder. *Nature.* **523**, 588-91. doi: 10.1038/nature14659. (2015).
Cordell, HJ. et al. International genome-wide meta-analysis identifies new primary biliary cirrhosis risk loci and targetable pathogenic pathways. *Nat Commun.* **6**, 8019. doi: 10.1038/ncomms9019. (2015).
Coronary Artery Disease (C4D) Genetics Consortium et al. A genome-wide association study in Europeans and South Asians identifies five new loci for coronary artery disease. *Nat Genet.* **43**, 339-44. doi: 10.1038/ng.782. (2011).
Evans, DM. et al. Interaction between ERAP1 and HLA-B27 in ankylosing spondylitis implicates peptide handling in the mechanism for HLA-B27 in disease susceptibility. *Nat Genet.* **43**, 761-7. doi: 10.1038/ng.873. (2011).
Fogh, I. et al. A genome-wide association meta-analysis identifies a novel locus at 17q112 associated with sporadic amyotrophic lateral sclerosis. *Hum Mol Genet.* **23**, 2220-31. doi: 10.1093/hmg/ddt587. (2014).
Gaulton, KJ. et al. Genetic fine mapping and genomic annotation defines causal mechanisms at type 2 diabetes susceptibility loci. *Nat Genet.* **47**, 1415-25. doi: 10.1038/ng.3437. (2015).
Genetic Analysis of Psoriasis Consortium & the Wellcome Trust Case Control Consortium 2 et al. A genome-wide association study identifies new psoriasis susceptibility loci and an interaction between HLA-C and ERAP1. *Nat Genet.* **42**, 985-90. doi: 10.1038/ng.694. (2010).
International Consortium for Systemic Lupus Erythematosus Genetics (SLEGEN) et al. Genome-wide association scan in women with systemic lupus erythematosus identifies susceptibility variants in ITGAM, PXK, KIAA1542 and other loci. *Nat Genet.* **40**, 204-10. doi: 10.1038/ng.81. (2008).
International Multiple Sclerosis Genetics Consortium et al. Genetic risk and a primary role for cell-mediated immune mechanisms in multiple sclerosis. *Nature.* **476**, 214-9. doi: 10.1038/nature10251. (2011).
International Stroke Genetics Consortium (ISGC) et al. Genome-wide association study identifies a variant in HDAC9 associated with large vessel ischemic stroke. *Nat Genet.* **44**, 328-33. doi: 10.1038/ng.1081. (2012).
Lambert, JC. et al. Meta-analysis of 74,046 individuals identifies 11 new susceptibility loci for Alzheimer's disease. *Nat Genet.* **45**, 1452-8. doi: 10.1038/ng.2802. (2013).
Liu, JZ. et al. Association analyses identify 38 susceptibility loci for inflammatory bowel disease and highlight shared genetic risk across populations. *Nat Genet.* **47**, 979-86. doi: 10.1038/ng.3359. (2015).

Mells, GF. et al. Genome-wide association study identifies 12 new susceptibility loci for primary biliary cirrhosis. *Nat Genet.* **43**, 329-32. doi: 10.1038/ng.789. (2011).
Moffatt, MF. et al. A large-scale, consortium-based genomewide association study of asthma. N Engl J Med. 363, 1211-21. doi: 10.1056/NEJMoa0906312. (2010).
Morris, AP. et al. Large-scale association analysis provides insights into the genetic architecture and pathophysiology of type 2 diabetes. *Nat Genet.* **44**, 981-90. doi: 10.1038/ng.2383. (2012).
Nair, RP. et al. Genome-wide scan reveals association of psoriasis with IL-23 and NF-kappaB pathways. *Nat Genet.* **41**, 200-204. doi: 10.1038/ng.311. (2009). (dbGaP accession number phs000019)
Okada, Y. et al. Genetics of rheumatoid arthritis contributes to biology and drug discovery. *Nature.* **506**, 376-81. doi: 10.1038/nature12873. (2014).
Otowa, T. et al. Meta-analysis of genome-wide association studies of anxiety disorders. *Mol Psychiatry.* **21**, 1391-9. doi: 10.1038/mp.2015.197. (2016).
Patsopoulos, NA. et al. Genome-wide meta-analysis identifies novel multiple sclerosis susceptibility loci. *Ann Neurol.* **70**, 897-912. doi: 10.1002/ana.22609. (2011).
Pattaro, C. et al. Genetic associations at 53 loci highlight cell types and biological pathways relevant for kidney function. *Nat Commun.* **7**, 10023. doi: 10.1038/ncomms10023. (2016).
Schunkert, H. et al. Large-scale association analysis identifies 13 new susceptibility loci for coronary artery disease. *Nat Genet.* **43**, 333-8. doi: 10.1038/ng.784. (2011). (www.CARDIOGRAMPLUSC4D.ORG)
Tobacco and Genetics Consortium et al. Genome-wide meta-analyses identify multiple loci associated with smoking behavior. *Nat Genet.* **42**, 441-7. doi: 10.1038/ng.571. (2010).
UK IBD Genetics Consortium et al. Genome-wide association study of ulcerative colitis identifies three new susceptibility loci, including the HNF4A region. *Nat Genet.* **41**, 1330-4. doi: 10.1038/ng.483. (2009).
UK Parkinson's Disease Consortium et al. Dissection of the genetics of Parkinson's disease identifies an additional association 5' of SNCA and multiple associated haplotypes at 17q21. *Hum Mol Genet.* **20**, 345-53. doi: 10.1093/hmg/ddq469. (2011).
Wellcome Trust Case Control Consortium et al. Genome-wide association study of 14,000 cases of seven common diseases and 3,000 shared controls. *Nature.* **447**, 661-78. doi: 10.1038/nature05911. (2007).

Supplementary Figures

A

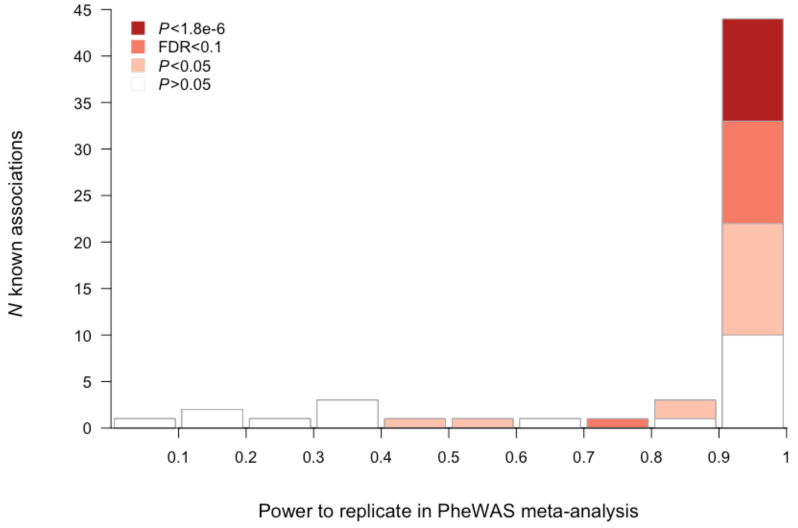

B

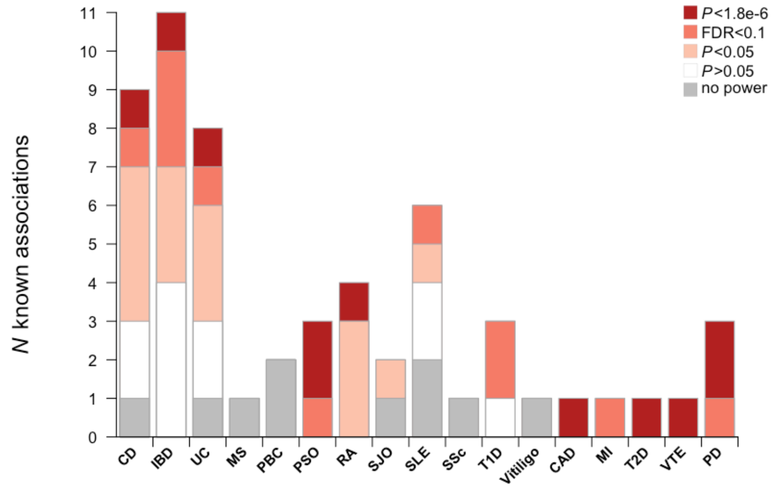

Supplementary Fig. 1. Replication of known GWAS associations through meta-analysis of PheWAS results from four disease-agnostic cohorts with extensive health information. (A) Distribution of replication rate based on statistical power to detect an association. Power to replicate at P<0.05 is shown. Power is calculated based on the SNP frequency, the direction and point estimate of effect size of the SNP in published studies, the number of cases and controls in the meta-analysis, and a disease prevalence of 1%. **(B)** Number of known GWAS associations that replicated or failed to replicate in the PheWAS meta-analysis, segregated by disease. The published Parkinson's disease (PD) GWAS included 23andMe results.

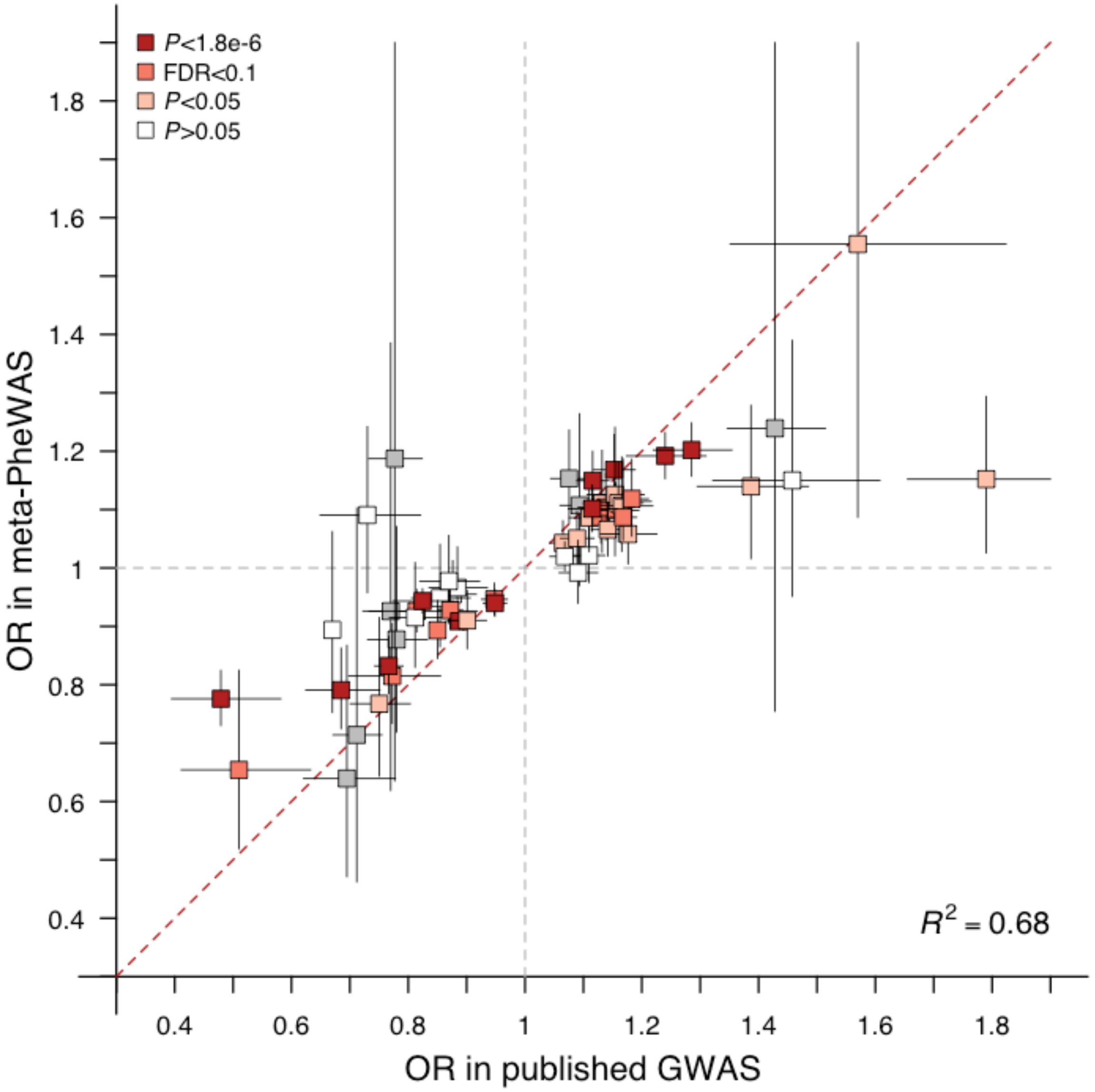

Supplementary Fig. 2. Effect sizes of known GWAS associations in the meta-PheWAS. Odds ratios (ORs) and 95% confidence intervals in the published GWAS are shown on the x axis. ORs and 95% confidence intervals in the meta-PheWAS are shown on the y axis. Powered associations (beta<0.8, alpha = 0.05) reaching $P<1.8\text{x}10^{-6}$ (Bonferroni corrected pvalue), FDR<0.1 or $P<0.05$ in meta-PheWAS are shown in dark red, red and light red respectively. Powered associations not reaching $P<0.05$ in meta-PheWAS are shown in white. Associations not powered in the meta-PheWAS are shown in grey. The regression coefficient R^2 between published ORs and ORs in meta-PheWAS is indicated.

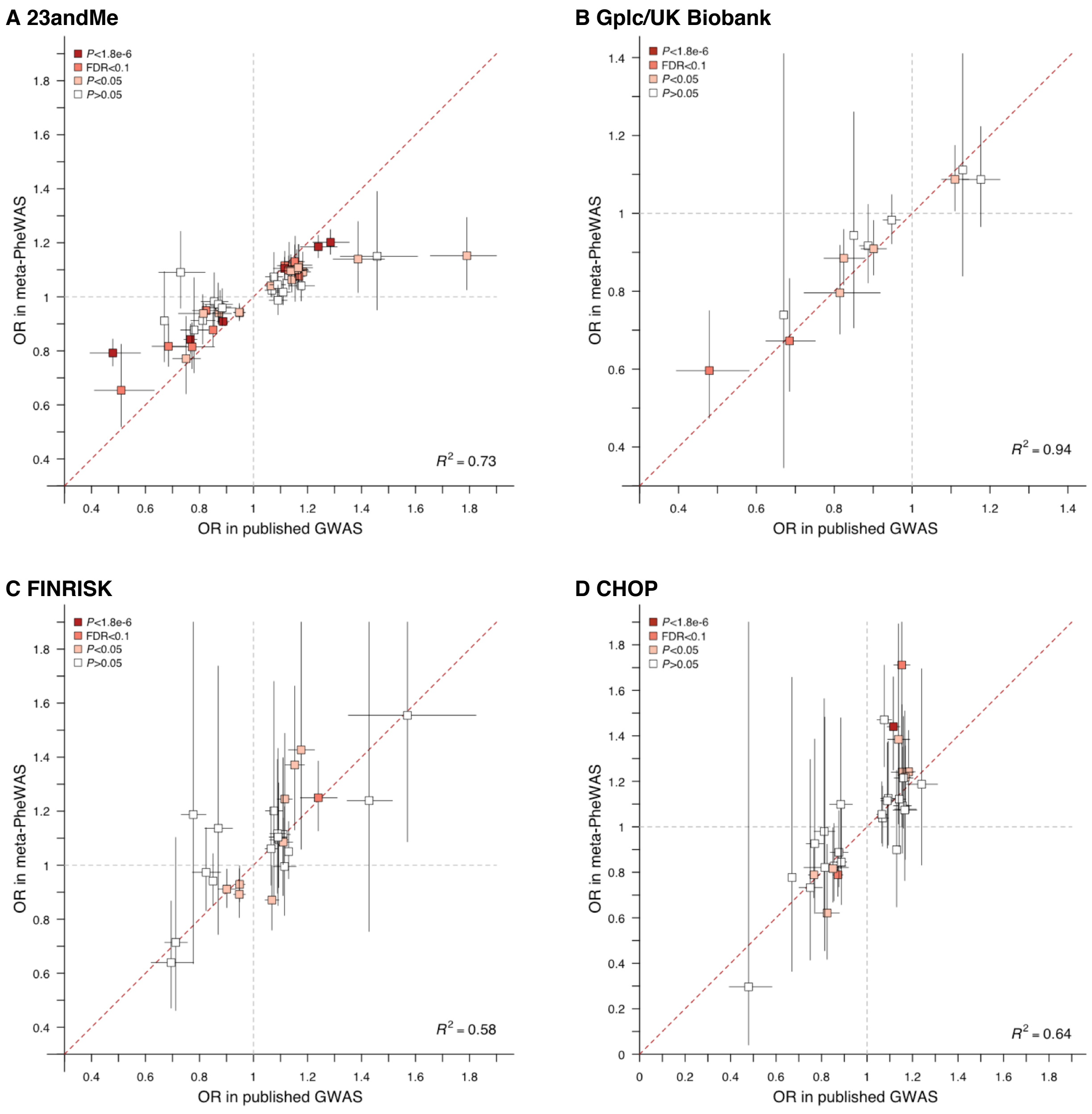

Supplementary Fig. 3. Effect sizes of known GWAS associations in the disease-agnostic cohorts PheWAS. Odds ratios (ORs) and 95% confidence intervals in the published GWAS are shown on the x axis. ORs and 95% confidence intervals in 23andMe (A), Gplc/UK Biobank (B), FINRISK (C) and CHOP (D) are shown on the y axis. Associations reaching $P<1.8\times10^{-6}$ (Bonferroni corrected pvalue), FDR<0.1 or $P<0.05$ in the PheWAS are shown in dark red, red and light red respectively. Associations not reaching $P<0.05$ are shown in white. The regression coefficients R^2 between published ORs and ORs in meta-PheWAS are indicated.

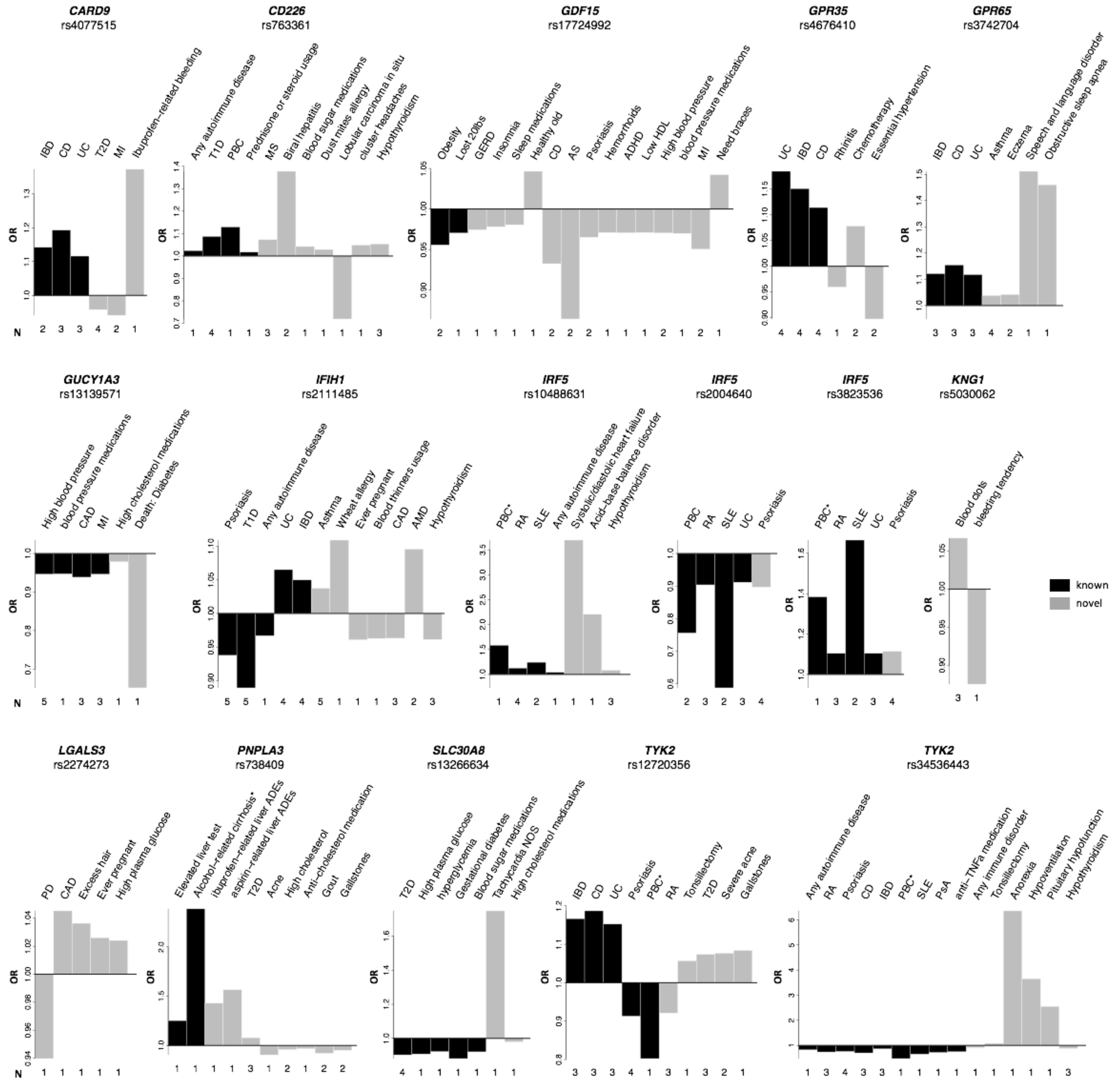

Supplementary Fig. 4. Effects of replicated and putative novel associations (FDR<0.1). Sixteen SNPs showed novel associations at FDR<0.1 (P<7e-4) in the meta-analysis of PheWAS and GWAS results. Effects (Odds ratio, OR) of the replicated known (black) and novel (grey) associations reaching FDR<0.1 are shown. The number (N) of disease-agnostic and GWAS cohorts contributing to a respective phenotype is indicated. *, Result derived from GWAS cohort only since the corresponding phenotype is not captured or not powered in the disease-agnostic cohorts tested.

A I^2<40%

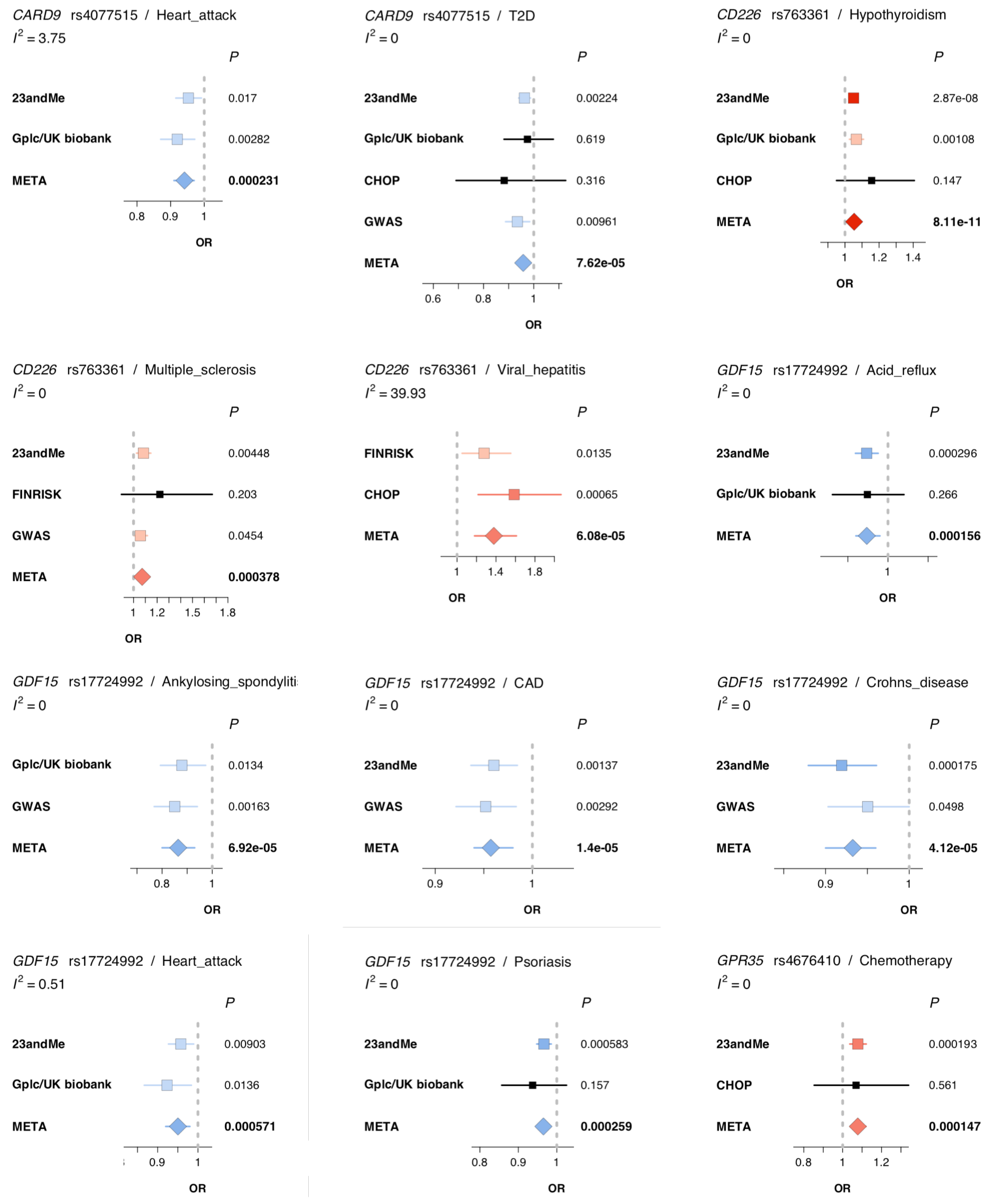

CARD9 rs4077515 / Heart_attack
I^2 = 3.75
P
23andMe 0.017
GpIc/UK biobank 0.00282
META 0.000231
0.8 0.9 1
OR
CARD9 rs4077515 / T2D
I^2 = 0
P
23andMe 0.00224
GpIc/UK biobank 0.619
CHOP 0.316
GWAS 0.00961
META 7.62e-05
0.6 0.8 1
OR
CD226 rs763361 / Hypothyroidism
I^2 = 0
P
23andMe 2.87e-08
GpIc/UK biobank 0.00108
CHOP 0.147
META 8.11e-11
1 1.2 1.4
OR
CD226 rs763361 / Multiple_sclerosis
I^2 = 0
P
23andMe 0.00448
FINRISK 0.203
GWAS 0.0454
META 0.000378
1 1.2 1.5 1.8
OR
CD226 rs763361 / Viral_hepatitis
I^2 = 39.93
P
FINRISK 0.0135
CHOP 0.00065
META 6.08e-05
1 1.4 1.8
OR
GDF15 rs17724992 / Acid_reflux
I^2 = 0
P
23andMe 0.000296
GpIc/UK biobank 0.266
META 0.000156
1
OR
GDF15 rs17724992 / Ankylosing_spondyliti
I^2 = 0
P
GpIc/UK biobank 0.0134
GWAS 0.00163
META 6.92e-05
0.8 1
OR
GDF15 rs17724992 / CAD
I^2 = 0
P
23andMe 0.00137
GWAS 0.00292
META 1.4e-05
0.9 1
OR
GDF15 rs17724992 / Crohns_disease
I^2 = 0
P
23andMe 0.000175
GWAS 0.0498
META 4.12e-05
0.9 1
OR
GDF15 rs17724992 / Heart_attack
I^2 = 0.51
P
23andMe 0.00903
GpIc/UK biobank 0.0136
META 0.000571
0.9 1
OR
GDF15 rs17724992 / Psoriasis
I^2 = 0
P
23andMe 0.000583
GpIc/UK biobank 0.157
META 0.000259
0.8 0.9 1
OR
GPR35 rs4676410 / Chemotherapy
I^2 = 0
P
23andMe 0.000193
CHOP 0.561
META 0.000147
0.8 1 1.2
OR

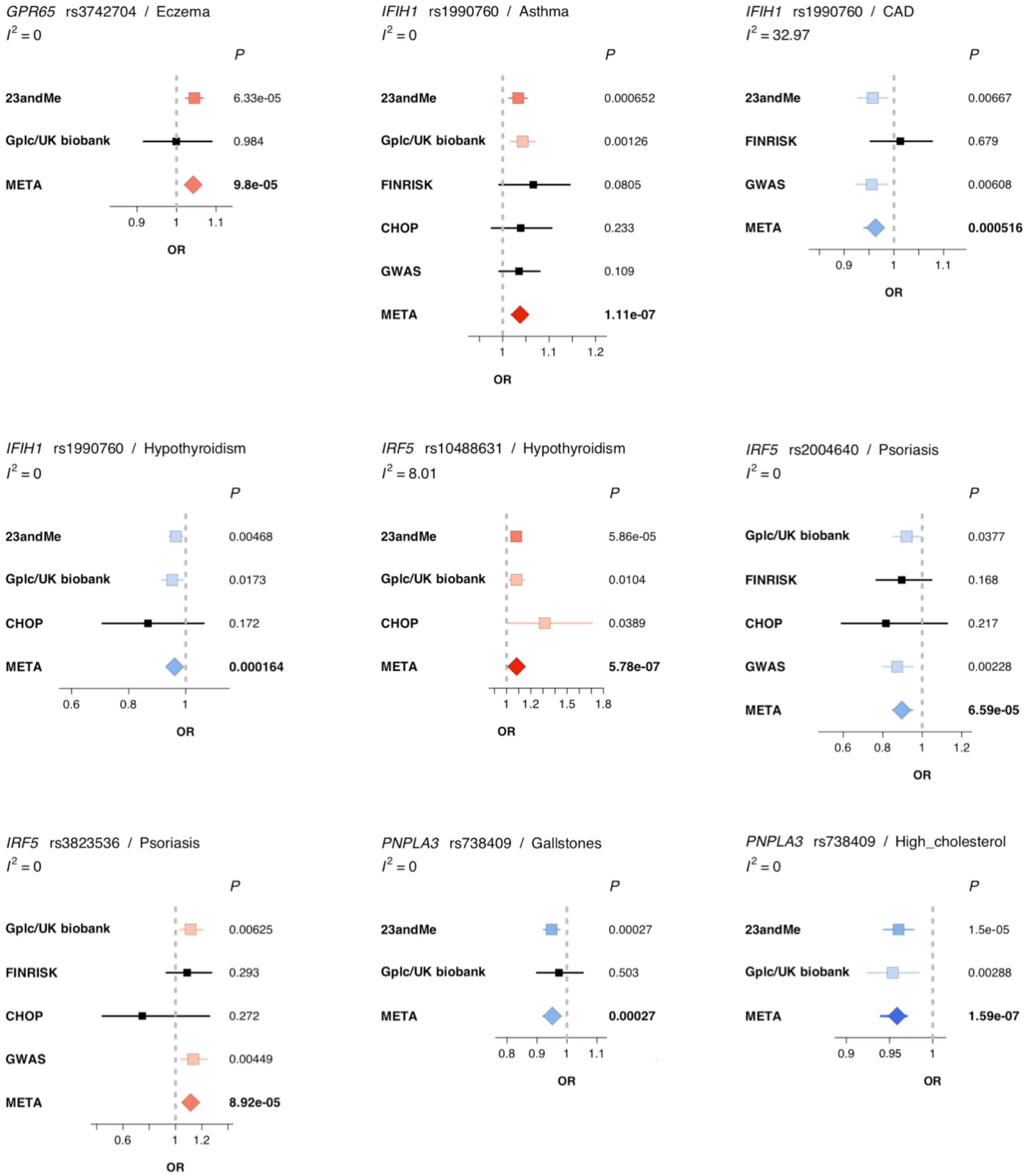
GPR65 rs3742704 / Eczema
$I^2 = 0$
P
23andMe 6.33e-05
Gplc/UK biobank 0.984
META 9.8e-05
0.9 1 1.1
OR
IFIH1 rs1990760 / Asthma
$I^2 = 0$
P
23andMe 0.000652
Gplc/UK biobank 0.00126
FINRISK 0.0805
CHOP 0.233
GWAS 0.109
META 1.11e-07
1 1.1 1.2
OR
IFIH1 rs1990760 / CAD
$I^2 = 32.97$
P
23andMe 0.00667
FINRISK 0.679
GWAS 0.00608
META 0.000516
0.9 1 1.1
OR
IFIH1 rs1990760 / Hypothyroidism
$I^2 = 0$
P
23andMe 0.00468
Gplc/UK biobank 0.0173
CHOP 0.172
META 0.000164
0.6 0.8 1
OR
IRF5 rs10488631 / Hypothyroidism
$I^2 = 8.01$
P
23andMe 5.86e-05
Gplc/UK biobank 0.0104
CHOP 0.0389
META 5.78e-07
1 1.2 1.5 1.8
OR
IRF5 rs2004640 / Psoriasis
$I^2 = 0$
P
Gplc/UK biobank 0.0377
FINRISK 0.168
CHOP 0.217
GWAS 0.00228
META 6.59e-05
0.6 0.8 1 1.2
OR
IRF5 rs3823536 / Psoriasis
$I^2 = 0$
P
Gplc/UK biobank 0.00625
FINRISK 0.293
CHOP 0.272
GWAS 0.00449
META 8.92e-05
0.6 1 1.2
OR
PNPLA3 rs738409 / Gallstones
$I^2 = 0$
P
23andMe 0.00027
Gplc/UK biobank 0.503
META 0.00027
0.8 0.9 1 1.1
OR
PNPLA3 rs738409 / High_cholesterol
$I^2 = 0$
P
23andMe 1.5e-05
Gplc/UK biobank 0.00288
META 1.59e-07
0.9 0.95 1
OR

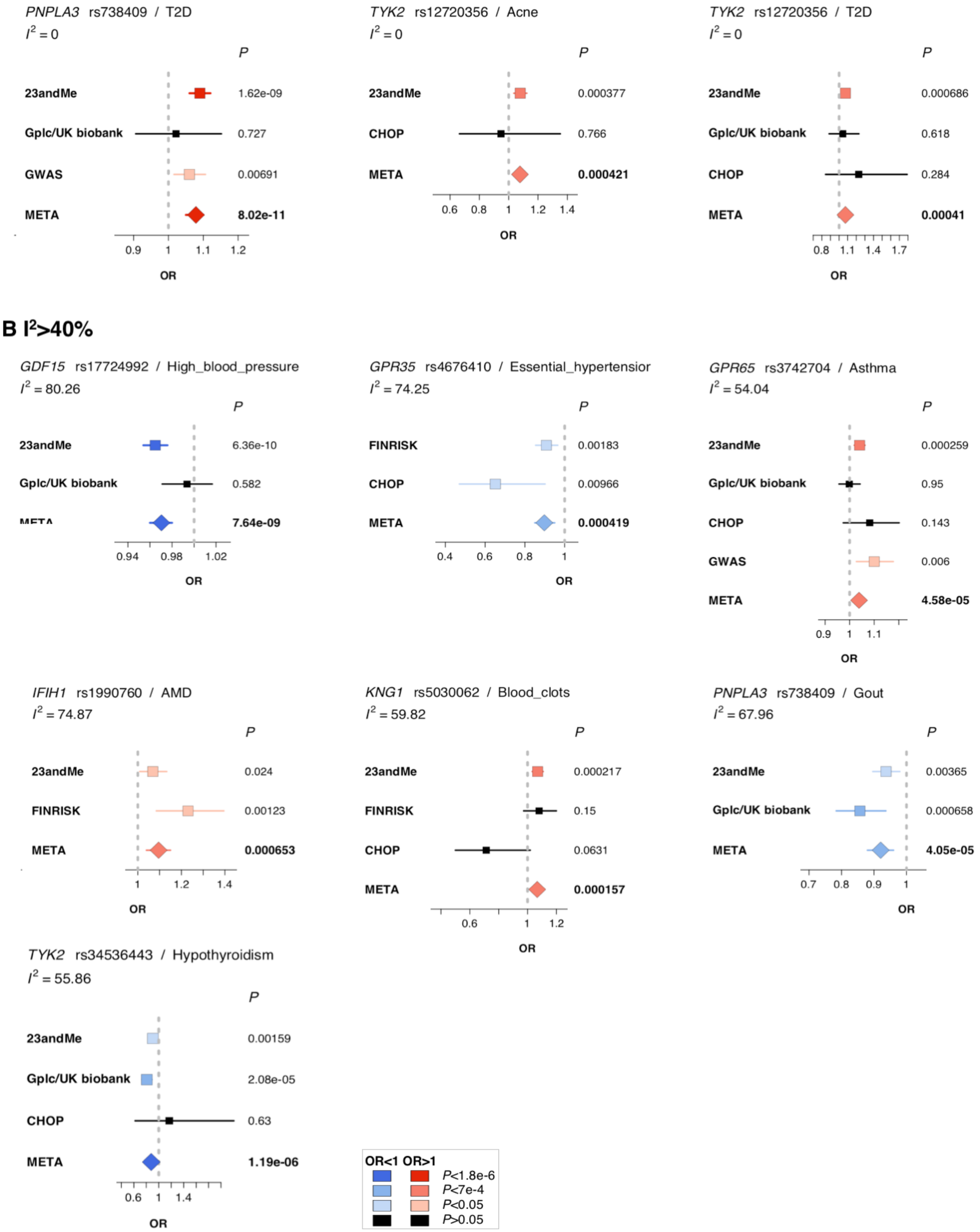

Supplementary Fig. 5. Effect sizes of putative novel associations observed in the individual cohorts and in the meta-analysis. 31 putative novel associations were obtained by meta-analyzing associations results from the disease-agnostic cohorts with GWAS results. Odds ratios (OR), 95% confidence intervals and *P* values are shown. **A.** Heterogeneity I^2 statistic <40%. **B.** I^2>40%.

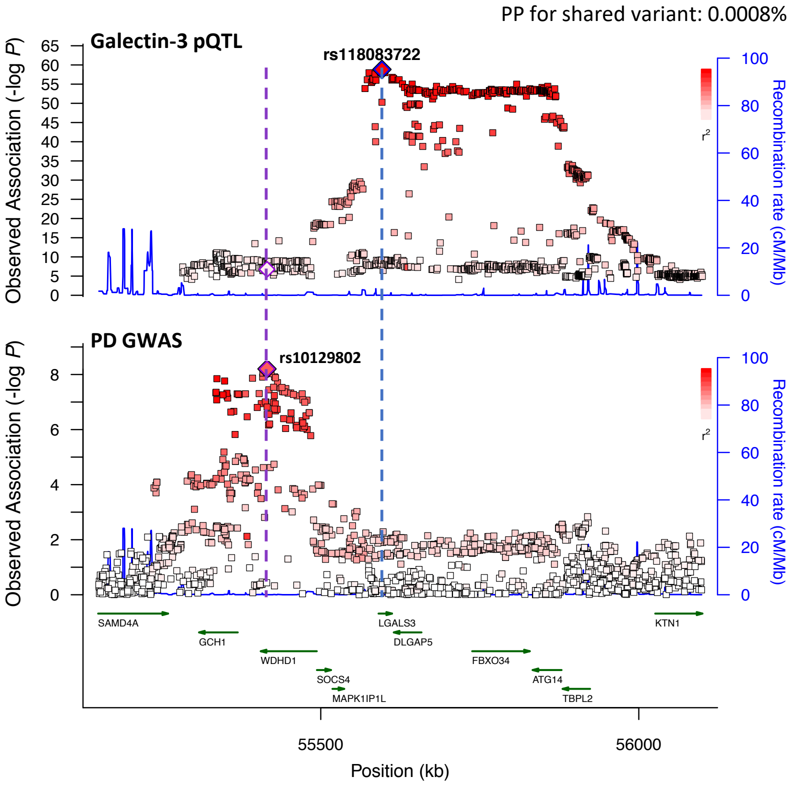

Supplementary Fig. 6. Independent associations for galectin-3 plasma levels and Parkinson's disease risk at the *GCH1-LGALS3* locus. Regional association results at the *GCH1-LGALS3* demonstrate that the associations with circulating plasma galectin-3 levels (top panel) and Parkinson's disease (PD, bottom panel) risk are driven by different causal SNPs. Co-localization test using *coloc* returns a posterior probability (PP) for a shared variant of 0.0008%.

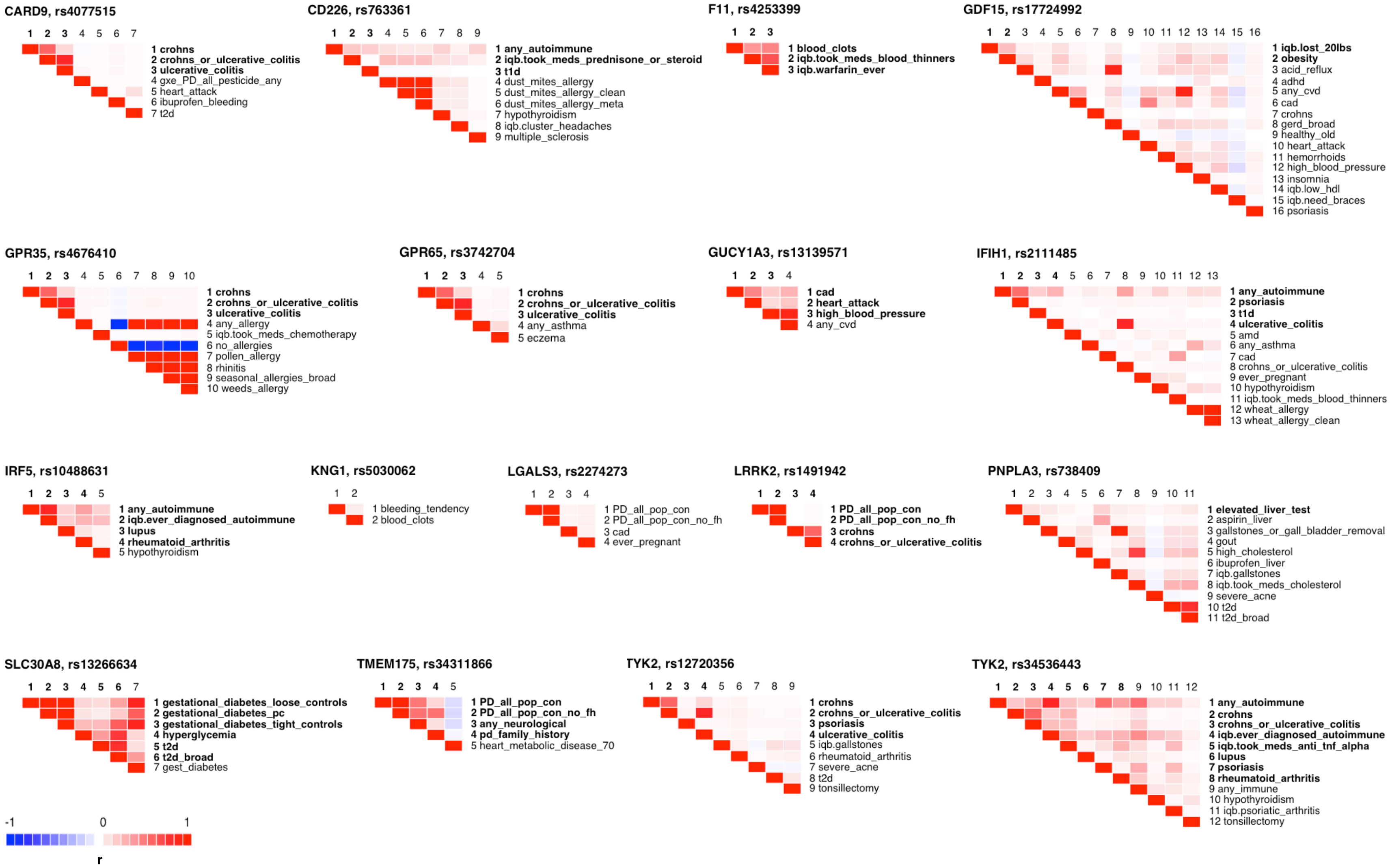

Supplementary Fig. 7. Phenotype-phenotype correlations in 23andMe. Pairwise phenotype correlation was calculated in participants for whom data on both phenotypes was available through r=cov/sqrt(var1*var2), with cov=covariance of phenotype 1 and phenotype 2, var1=variance in phenotype 1, var2=variance of phenotype 2. Only phenotypes associated at FDR<0.1 with indicated SNPs are shown. Phenotypes related to known associations are highlighted in bold.

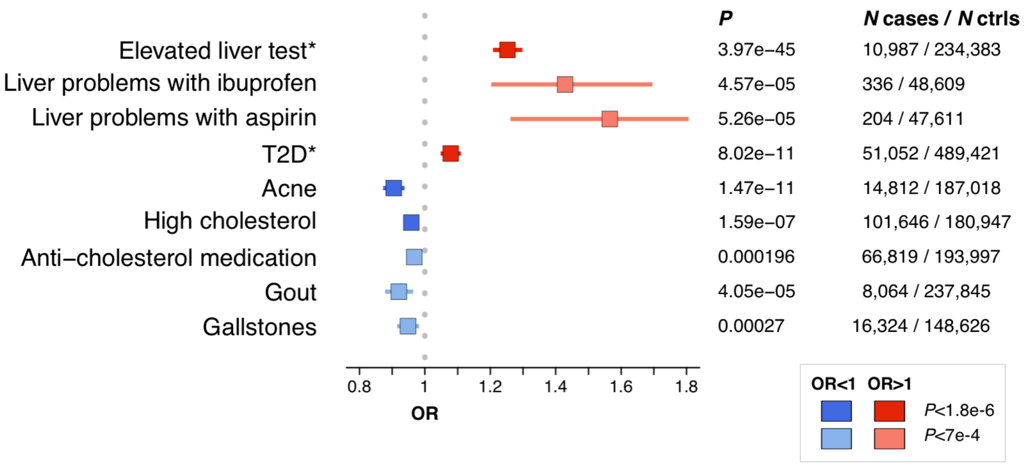

Supplementary Fig. 8. Association of *PNPLA3* rs738409-G with multiple disease-relevant endpoints. Associations reaching FDR<0.1 in joint meta-analysis of PheWAS results with available GWAS results are shown. Odds ratios, 95% confidence intervals, *P* values, and numbers (*N*) of cases and controls are represented. Known GWAS associations are indicated with an asterix (*).

Supplementary Tables

Supplementary Table 1. SNP-phenotype associations reported earlier in published GWAS for the 25 SNPs interrogated through PheWAS in this study

Reported Gene	SNP	Phenotype$	BETA^	*P* value	Phenotype testable in PheWAS	Reference
ATG16L1	rs13391356	Crohn's disease	-0.211	4.2e-70	yes	3
		IBD	-0.136	5.9e-35	yes	3
CARD9	rs11145766	IBD	-0.194	4.1e-22	yes	3
		Crohn's disease	-0.192	7.5e-19	yes	3
		Ulcerative colitis	-0.141	1.9e-11	yes	3
CARD9	rs4077515	Crohn's disease	0.168	3.23e-43	yes	36
		IBD	0.155	1.27e-53	yes	36
		Ulcerative colitis	0.132	1.02e-25	yes	36
CD226	rs763361	T1D	0.122	1.8e-11	yes	14
		IBD	0.0558	4.7e-09	yes	36
		Mean platelet volume*	-0.007	3.36e-11		15
CDHR3	rs6967330	Asthma (childhood onset)	0.231	3e-14	yes	52
F11	rs4253399	aPTT*	-0.48	6e-43		56
		VTE	0.215	2e-14	yes	57
		FXI levels*	0.088	6.22e-08		55
F12	rs2731672	aPTT*	1.55	8.9e-60		56
		FXII levels *	NA	1e-7		63
GDF15	rs17724992	BMI *	-0.019	3.4e-08		65
GPR35	rs4676410	Ulcerative colitis	0.137	2.1e-18	yes	3
		IBD	0.129	8.1e-19	yes	3
		Crohn's disease	0.095	3.2e-08	yes	3
GPR65	rs3742704	Crohn's disease	0.155	9.41e-14	yes	36
		IBD	0.144	7.29e-17	yes	36
		Ulcerative colitis	0.129	2.16e-09	yes	36
GUCY1A3	rs13139571	Blood pressure **	-0.26	2.2e-10	yes **	68
		CAD	-0.052	9.5e-07	yes	83
		MI	-0.053	5.8e-06	yes	83
IFIH1	rs2111485	IgAD	0.67	7.3e-10		84
		T1D	-0.162	3.8e-18	yes	14
		Vitiligo	-0.261	4.9e-15	yes	85
		IBD	0.0629	4.6e-09	yes	36
		Ulcerative colitis	0.0854	2.11e-10	yes	36
		SLE	-0.139	1.27e-11	yes	35
		Psoriasis	-0.131	2.7e-08	yes	47
IRF5	rs10488631	SLE	0.582	9.4e-45	yes	35
		Primary biliary cirrhosis	0.464	5.1e-23	yes	40
		RA	0.163	1.3e-11	yes	38
		Systemic scleroderma	0.425	1e-10	yes	41
		Sjogren's syndrome	0.451	5.4e-16	yes	39
IRF5	rs2004640	Primary biliary cirrhosis	-0.198	4.1e-19	yes	40

		RA	-0.104	4.3e-10	yes	38
		SLE	-0.328	2.9e-31	yes	35
		Sjogren's syndrome	-0.314	2.9e-14	yes	39
IRF5	rs3823536	SLE	0.356	5e-36	yes	35
		Primary biliary cirrhosis	0.199	4.1e-19	yes	40
		RA	0.104	2.4e-11	yes	38
		Ulcerative colitis	0.095	4.4e-14	yes	3
KNG1	rs5030062	aPTT *	-1.19	1.1e-185		56
		FXI levels*	0.083	1.19e-07		55
LGALS3	rs2274273	galectin-3 plasma levels*	0.185	2.35e-188		71
LRRK2	rs11564187	Crohn's disease	0.377	6.80e-26	yes	3
		IBD	0.327	3.30e-20	yes	3
LRRK2	rs1491942	Parkinson's disease	0.239	6.00e-14	yes	80
		IBD	0.104	1.80e-17	yes	36
		Crohn's disease	0.124	3.51e-17	yes	36
		Ulcerative colitis	0.087	1.56e-08	yes	36
PNPLA3	rs738409	NAFLD	1.18	3.6e-43		73
		Alcohol-related cirrhosis	0.784	2e-48		86
		CT hepatic steatosis *	0.26	4.3e-34		73
		ALT **	0.06	1e-45	yes**	72
SLC30A8	rs13266634	Fasting glucose *	-0.029	1.47e-35		87
		T2D	-0.119	1.3e-18	yes	88
TMEM175	rs34311866	Parkinson's disease	0.239	1e-43	yes	80
TMEM175	rs34884217	Parkinson's disease	-0.301	1.6e-12	yes	80
TYK2	rs12720356	Crohn's disease	0.1433	3.39e-11	yes	36
		IBD	0.1483	4.13e-16	yes	36
		Psoriasis	-0.287	4e-11	yes	47
		Ulcerative colitis	0.1533	1.67e-11	yes	36
		SLE	-0.314	5.1e-09	yes	35
TYK2	rs34536443	Primary biliary cirrhosis	-0.653	1.2e-12	yes	40
		Psoriasis	-0.634	9.1e-31	yes	47
		RA	-0.378	4.6e-16	yes	38
		Multiple sclerosis	-0.248	1.8e-14	yes	48
		SLE	-0.673	2e-11	yes	35
		T1D	-0.4	4.4e-15	yes	14
		IBD	-0.2087	1.83e-13	yes	36
		Crohn's disease	-0.2872	1.86e-16	yes	36

$ Significant associations (*P*<5e-8) in the European population
^ Effect of minor allele
* Quantitative traits
** Quantitative traits related to binary traits tested in PheWAS: blood pressure and high blood pressure, ALT and elevated liver test
IBD, inflammatory bowel disease; T1D, type 1 diabetes; aPTT, activated partial thromboplastin time; VTE, venous thromboembolism; BMI, body mass index; CAD, Coronary Artery Disease; MI, myocardial Infarction; IgAD, immunoglobulin A Deficiency; SLE, Systemic Lupus Erythematosus; RA, rheumatoid arthritis; NAFLD, Non-alcoholic fatty liver disease; ALT, alanine aminotransferase

Supplementary Table 2. Examples of mapped phenotypic endpoints

Phenotype	Meta-analyzed mapped endpoints	Cohort-specific / unmapped endpoints
Heart attack	23andMe - “heart attack”	23andMe - "early onset heart attack"
	Gplc UK Biobank - “Heart attack”	FINRISK - "Myocardial infarction"
	FINRISK - “IPHG Myocardial infarction”	FINRISK - "Myocardial infarction, strict"
High blood pressure	23andMe - "high blood pressure"<br>UK Biobank - "Hypertension"	23andMe - "iqb.blood pressure meds"
	FINRISK - “IPHG Hypertension”<br>CHOP - "Hypertension"	CHOP - "Elevated blood pressure reading"
Gall stones	23andMe - "iqb.gallstones"	23andMe - "gallstones or gall bladder removal"
	Gplc UK Biobank - "Gall stones"	CHOP - "Cholelithiasis and cholecystitis"
	CHOP - "Cholelithiasis"	CHOP - "Cholelithiasis with other cholecystitis"

Full list of individual phenotypic endpoints that are mapped for meta-analysis is provided in Supplementary Data file 1.

Supplementary Table 3. Mapped phenotypes found in at least two disease-agnostic cohorts and included in the meta-analysis

	N cases/N controls			
**Phenotype**	**23andMe**	**Gplc UK Biobank**	**FINRISK**	**CHOP**
1. Autoimmune diseases				
Ankylosing spondylitis	NA	1026/111311	119/21173	91/13333
Celiac disease	6611/495405	NA	143/21149	151/9748
Crohn's disease	1867/251313	NA	95/21197	577/9748
IBD	5495/246251	NA	430/20862	663/9748
SLE	1336/246359	NA	43/21249	NA
Multiple sclerosis	1399/249152	NA	80/21212	NA
Psoriasis	16001/242065	1304/111033	325/20967	91/12699
Rheumatoid arthritis	6568/249421	1328/111009	1374/19918	NA
T1D	1607/247438	97/112238	862/20430	239/13377
Ulcerative colitis	4069/250210	NA	257/21035	205/9748
Vitiligo	963/419015	NA	NA	56/12934
2. Blood diseases				
Abnormal coagulation profile	NA	NA	21/21271	41/13024
Anemia	13560/72539	197/112140	NA	NA
Blood in stool	7113/91512	NA	NA	111/11360
Coagulation defects	NA	NA	56/21236	255/13024
Congenital coagulation defects	NA	NA	20/21272	97/13024
Primary thrombocytopenia	NA	NA	26/21266	72/13024
thrombocytopenia	NA	NA	69/21223	174/13024
3. Cancers				
Any cancer	67170/485702	NA	2679/18613	100/13104
Basal cell carcinoma	14480/258540	1034/111303	NA	NA
Bladder cancer	828/274338	NA	136/21156	NA
Brain cancer	NA	NA	55/21237	124/13601
Breast cancer	5510/133058	1843/34552	668/20624	NA
Cervical cancer	NA	402/58556	29/21263	NA
Chemotherapy	15424/515350	NA	NA	252/13104
Colorectal cancer	1537/276305	158/112179	259/21033	NA
Lipoma	21238/127699	NA	NA	21/13710
Lymphoid leukemia	NA	NA	50/21242	107/13613
Myeloproliferative neoplasm	1978/636034	NA	124/21168	22/13613
Non-Hodgkins lymphoma	741/272275	NA	143/21149	36/13613
Prostate cancer	4492/155380	833/52270	515/20777	NA
Skin cancer	23439/240977	NA	811/20481	NA
Thyroid cancer	1270/274127	NA	78/21214	NA

4. Cardiovascular diseases				
Angina	5394/239601	3750/108141	NA	NA
CVD	81672/183219	NA	4279/17013	NA
Atrial fibrillation	7764/219788	874/111463	1801/19491	NA
Blood clots	14254/499076	NA	762/20530	70/13468
CAD	9630/237947	NA	3336/17956	NA
Cardiomyopathy	NA	NA	223/21069	63/13681
Deep vein thrombosis	NA	NA	203/21089	24/13540
Essential hypertension	NA	NA	4032/17260	160/13408
Heart attack	5499/246683	2786/109162	1391/19901	NA
Heart failure	1841/243366	NA	2018/19274	65/13104
Heart valve disorders	1399/43647	802/111535	850/20442	120/12830
High blood pressure	121891/385876	29847/80466	4178/17114	202/13408
Hypertensive heart and/or renal disease	NA	NA	492/20800	51/13408
Primary/intrinsic cardiomyopathies	NA	NA	166/21126	54/13681
Stroke	5479/508263	1571/110147	1907/19385	NA
Varicose veins	40949/121550	NA	NA	47/13468
5. Diseases of digestive system				
Acid reflux	73710/177745	5073/107264	NA	NA
Appendicitis	29169/178759	NA	NA	62/13735
Constipation	1453/97369	NA	NA	1178/9748
Diverticulitis	3077/415429	1327/111010	NA	NA
Gallstones	25838/244204	1884/110142	NA	89/13636
GERD	130860/385247	NA	NA	1758/10420
Hemorrhoids	60335/104379	NA	NA	23/13468
Irritable bowel syndrome	48921/419376	2774/109563	NA	116/9748
Ulcer	6312/80102	822/111515	NA	NA
6. Endocrine and metabolic diseases				
Diabetes mellitus	NA	NA	3234/18058	371/13377
Gout	6452/127120	1612/110725	NA	NA
High cholesterol	177886/160668	14678/97348	NA	48/13546
Hyperlipidemia	NA	NA	1142/20150	143/13546
Hyperthyroidism	3638/279816	881/111456	NA	NA
Hypoglycemia	NA	NA	77/21215	57/12400
Hypothyroidism	17431/116070	5517/106820	NA	232/13261
Insulin user	1649/86695	NA	NA	26/13377
Obesity	15297/69248	NA	579/20713	392/12645
Morbid obesity	NA	NA	579/20713	34/12645
T2D	27123/525417	784/111522	1615/19677	159/13377

7. Diseases of the eye				
AMD	2569/67156	NA	521/20771	NA
Astigmatism	110013/104519	NA	NA	98/12992
Cataract	11500/76639	1820/110206	NA	71/13775
Farsightedness	49315/115664	NA	NA	71/12992
Glaucoma	2413/114683	1275/111062	NA	31/13593
Nearsightedness	185123/140366	NA	NA	71/12992
Strabismus	7150/158339	NA	NA	467/12549
8. Genitourinary diseases				
Chronic kidney disease	1341/85814	NA	207/21085	55/13158
End stage renal disease	NA	NA	203/21089	22/13158
Kidney stones	13486/119730	890/111447	NA	99/13486
Renal failure	NA	NA	174/21118	95/13158
Renal failure NOS	NA	NA	60/21232	22/13158
9. Allergies and related immune diseases				
Acute upper respiratory infections	NA	NA	1560/19732	2714/10032
Allergic conjunctivitis	NA	NA	70/21222	118/12063
Allergies,other	NA	NA	130/21162	836/9247
Asthma	28400/126511	14038/98299	3233/18059	3132/10276
Atopic/contact dermatitis due to other or unspecified	NA	NA	82/21210	1315/9301
Drug allergy	9267/26571	NA	41/21251	509/11385
Eczema	31625/218433	3134/109203	NA	NA
Food allergy	13947/113514	NA	NA	1227/9247
Hayfever	8105/19984	6549/105788	454/20838	1981/8301
Penicillin allergy	25559/206265	NA	NA	273/11385
10. Infections				
Bacterial meningitis	853/82449	448/111889	NA	25/13699
Chronic hepatitis	NA	NA	92/21200	76/10977
Hepatitis C	1372/211221	NA	NA	21/10977
Herpes simplex	56979/105405	NA	742/20550	34/10977
Influenza	NA	NA	195/21097	45/11564
Lyme disease	4481/147202	NA	NA	38/13101
Measles	36593/45161	424/111913	503/20789	NA
Mononucleosis	17691/68127	125/112212	232/21060	31/10977
Mumps	29912/51724	263/112074	944/20348	NA
Myringotomy	3961/81302	NA	64/21228	NA
Pneumonia	39770/86836	1581/110756	46/21246	697/11642
Rubella	11538/68374	148/112189	NA	NA
Scarlet fever	6590/109517	137/112200	NA	NA
Septicemia	NA	78/112259	NA	58/13054

Shingles	16593/114894	NA	590/20702	NA
Tonsillitis	NA	753/111584	NA	2110/8301
Tuberculosis	966/164459	536/111801	NA	NA
Urinary tract infection	NA	NA	530/20762	356/12903
Viral hepatitis	NA	NA	204/21088	127/10977
11. Musculoskeletal diseases				
Back pain	15639/82392	19558/8920	NA	71/13658
Osteoarthritis	28428/135127	10063/102274	NA	NA
Osteoporosis	12210/152336	1788/110238	NA	36/13430
Scoliosis	8484/89567	NA	NA	858/12115
12. Neurological disorder				
Alzheimer's disease	920/221800	NA	703/20589	NA
Epilepsy	2624/242873	537/63579	558/20734	576/11787
Essential tremor	594/307890	NA	104/21188	NA
Migraine	102826/439398	3310/109027	545/20747	452/12345
Migraine with aura	10550/221352	NA	249/21043	NA
Parkinson's disease	11707/650723	NA	478/20814	NA
Restless leg syndrome	7510/74539	NA	43/21249	NA
13. Diseases of pregnancy				
Gestational diabetes	8005/148993	NA	313/20979	NA
14. Psychiatric diseases				
Anxiety	39812/202001	61430/50906	621/20671	404/10957
Autism	1939/498356	NA	NA	540/10790
Bipolar disorder	12537/504469	NA	154/21138	54/12618
Depression	59179/190660	6900/105437	1328/19964	123/12618
Eating disorder	11019/452125	NA	NA	70/12618
Mania	3103/112412	2081/34001	NA	NA
Obsessive-compulsive disorder	13025/483904	NA	NA	39/12618
Schizophrenia	965/500850	NA	292/21000	NA
15. Reproductive disorders				
Endometriosis	12545/76786	930/58081	NA	NA
Female infertility	12269/45334	NA	447/20845	NA
Male infertility	6464/37139	NA	52/21240	NA
PCOS	8053/135218	NA	NA	35/12782
Uterine fibroids	22410/67855	1687/57322	NA	NA
16. Respiratory diseases				
Bronchiectasis	NA	NA	531/20761	42/10276
Bronchitis	NA	NA	673/20619	48/10276
Chronic bronchitis	9457/75371	NA	83/21209	23/10276
Chronic sinusitis	NA	683/111654	NA	366/8301

COPD	2312/12084	NA	681/20611	76/10276
17. Sensation				
Hearing loss	15515/84737	28414/79588	NA	1693/11301
Tinnitus	36144/142221	212/112125	NA	NA
18. Skin diseases				
Acne	14812/187018	NA	48/21244	231/13040
19. Sleep disorders				
Insomnia	69612/184284	NA	NA	21/11795
Sleep apnea	21109/159458	NA	NA	659/11795

Full list of individual phenotypic endpoints that are mapped for meta-analysis is provided in Supplementary Data file 1. NOS, not otherwise specified

Supplementary Table 4. Replication of known GWAS associations in the PheWAS meta-analysis

			meta-PheWAS association results						Statistical power in meta-PheWAS#		
Reported gene	SNP	Known associated phenotype	*N* cases	*N* controls	OR^	*P* value	FDR	Direction	alpha= 0.05	alpha= 3.8e-4	alpha= 1.8e-6
ATG16L1	rs13391356	Crohn's disease	2353	259762	0.83	9.47E-10	**1.25e-6**	-??-	1	**1**	0.99
		IBD	6062	254700	0.93	5.40E-05	**0.0241**	-??-	1	**1**	1
CARD9	rs11145766	Crohn's disease	2444	261061	0.95	0.263	0.94	-??-	0.98	0.68	0.23
		IBD	6158	255999	0.95	0.122	0.857	-??-	1	**1**	0.95
		Ulcerative colitis	4274	259958	0.97	0.334	0.957	-??+	0.97	0.64	0.19
CARD9	rs4077515	Crohn's disease	2352	259728	1.12	1.90E-04	**0.0579**	+??+	1	**1**	0.95
		IBD	6061	254666	1.09	8.64E-06	**0.00497**	+??+	1	**1**	1
		Ulcerative colitis	4267	258625	1.07	0.00407	0.3656	+??+	1	**1**	0.97
CD226	rs763361	IBD	13616	526792	1.02	0.115	0.849	+?-+	1	**0.98**	0.78
		Type 1 diabetes	4572	655360	1.09	8.29E-05	**0.0322**	+++-	1	**0.99**	0.86
F11	rs4253399	Blood clots	7487	273305	1.19	3.50E-25	**1.93e-21**	+?++	1	**1**	1
GPR35	rs4676410	Crohn's disease	2530	282288	1.15	5.75E-05	**0.0251**	+?++	0.79	0.21	0.02
		IBD	6462	277008	1.15	2.05E-10	**2.98e-7**	+?++	1	**0.99**	0.88
		Ulcerative colitis	4525	281021	1.17	1.50E-09	**1.89e-6**	+?++	1	**0.96**	0.7
GPR65	rs3742704	Crohn's disease	5770	517318	1.11	8.52E-04	0.148	+??+	0.99	**0.8**	0.35
		IBD	13191	505971	1.1	1.28E-05	**0.00668**	+??+	1	**1**	0.97
		Ulcerative colitis	8627	514874	1.1	1.91E-04	**0.0579**	+??+	1	**0.91**	0.54
GUCY1A3	rs13139571	CAD	21918	505836	0.94	6.23E-07	**4.30e-4**	-?-?	1	**0.92**	0.58
		Heart attack	14298	632007	0.95	1.78E-04	**0.0551**	---?	0.98	0.68	0.23
		IBD	6492	275563	1.04	0.0204	0.63	+?++	0.94	0.47	0.1
		Psoriasis	17690	385065	0.94	2.35E-07	**1.84e-4**	----	1	**1**	1
		SLE	1379	267608	0.98	0.56	0.984	-?+?	0.95	0.52	0.12
		Type 1 diabetes	2785	391708	0.89	6.54E-05	**0.027**	----	1	**0.99**	0.88
IFIH1	rs2111485	Ulcerative colitis	4430	279790	1.06	0.0231	0.65	+?++	0.97	0.64	0.2
		Vitiligo	51	11216	0.93	0.708	0.994	???-	0.26	0.01	<0.01

		PBC	20	21272	NA	NA	NA	NA	0.14	<0.01	<0.01
IRF5	rs10488631	Rheumatoid arthritis	8031	381587	1.06	0.0251	0.657	+++?	1	**1**	0.92
		Sjogren's syndrome	89	21203	1.55	0.0207	0.634	??+?	0.44	0.04	<0.01
		SLE	1336	246359	1.15	0.0188	0.61	+???	1	**1**	1
		Systemic Scleroderma	18	21274	NA	NA	NA	NA	0.12	<0.01	<0.01
IRF5	rs2004640	PBC	20	21272	1.19	0.59	0.986	??+?	0.1	<0.01	<0.01
		Rheumatoid arthritis	2702	130927	0.91	0.00077	0.142	?--?	0.96	0.59	0.16
		Sjogren's syndrome	89	21203	0.64	0.0035	0.339	??-?	0.56	0.07	<0.01
		SLE	43	21249	0.71	0.125	0.857	??-?	0.33	0.02	<0.01
		Rheumatoid arthritis	2702	130927	1.09	0.00335	0.338	?++?	0.97	0.6	0.17
		SLE	32	21260	1.24	0.399	0.967	??+?	0.3	0.02	<0.01
IRF5	rs3823536	Ulcerative colitis	456	29480	1.11	0.132	0.862	??++	0.3	0.02	<0.01
LRRK2	rs11564187	Crohn's disease	1867	251313	1.15	0.157	0.886	+???	0.9	0.38	0.06
		IBD	5495	246251	1.14	0.0287	0.668	+???	1	**0.89**	0.51
LRRK2	rs1491942	Crohn's disease	1867	251313	1.12	0.00949	0.514	+???	0.83	0.26	0.03
		IBD	5834	267204	1.02	0.367	0.962	+?+?	0.99	0.77	0.32
		Parkinson's disease	9786	324827	1.1	1.51E-07	**1.39e-4**	+?-?	1	**1**	1
		Ulcerative colitis	4326	271245	0.99	0.77	0.998	-?+?	0.87	0.32	0.05
SLC30A8	rs13266634	Type 2 diabetes	48015	556969	0.91	5.04E-35	**4.64e-31**	--?-	1	**1**	1
TMEM175	rs34311866	Parkinson's disease	9308	297840	1.2	8.04E-21	**2.77e-17**	+???	1	**1**	1
TMEM175	rs34884217	Parkinson's disease	2162	323427	0.81	8.10E-05	**0.0322**	-???	1	**0.97**	0.72
TYK2	rs12720356	Crohn's disease	2353	259763	1.13	0.0175	0.599	+??+	0.84	0.27	0.03
		IBD	6062	254701	1.11	7.73E-04	0.142	+??+	1	**0.91**	0.54
		Psoriasis	17386	364096	0.93	1.27E-04	**0.0445**	--?-	1	**1**	1
		SLE	1336	246359	1.1	0.196	0.91	+???	1	**0.91**	0.55
		Ulcerative colitis	4268	258660	1.11	0.0066	0.446	+??+	0.99	0.75	0.29
TYK2	rs34536443	Crohn's disease	2333	259290	0.77	0.00317	0.329	-??-	0.92	0.42	0.08
		IBD	6038	254228	0.92	0.076	0.811	-??-	0.97	0.63	0.19
		Multiple sclerosis	1399	249152	0.88	0.19	0.907	-???	0.61	0.1	<0.01
		Psoriasis	17382	363480	0.78	2.58E-16	**5.47e-13**	--?-	1	**1**	1

Rheumatoid arthritis	7896	360430	0.8	1.25E-07	**1.19e-4**	--??	1	**1**	1
SLE	1336	246359	0.65	1.25E-04	**0.0442**	-???	1	**0.99**	0.9
Type 1 diabetes	1912	370633	0.89	0.203	0.914	--?-	0.99	0.76	0.3

^ Effect of minor allele. Refer to Supplementary Data file 1 for detailed information.
$ Direction of effect in 23andMe, Genomics plc UK Biobank, FINRISK, and CHOP, respectively.
# Statistical power to detect an association in the meta-PheWAS was calculated based on the effect allele frequency in the 1000Genomes European population, the published effect size in GWAS, the number of cases and controls in the meta-PheWAS and the corresponding disease prevalence reported by the Centers for Disease Control and Prevention (see Methods). Three significance cutoffs were tested: $P<0.05$, FDR<0.1 ($P<3.8e-4$), and Bonferroni-corrected significance ($P<1.8e-6$).
CAD, coronary artery disease; IBD, inflammatory bowel disease; SLE, systemic lupus erythematosus; PBC, primary biliary cirrhosis.

Supplementary Table 5. Putative novel associations identified in the meta-PheWAS

Reported Gene	SNP	Phenotype*	OR^	(CI95)	*P* value	*N* cohorts	*N* cases	*N* controls	FDR	Direction$	I^2
A. Known autoimmunity-associated SNPs											
CARD9	rs4077515	Heart attack	0.94	(0.91-0.97)	0.00023	2	8285	355845	0.0453	--???	3.75
		Ibuprofen AE: bleeding	1.37	(1.16-1.64)	4.51E-04	1	248	48697	0.0759	+????	NA
		T2D	0.96	(0.94-0.98)	7.62E-05	4	51194	500981	0.0194	--?--	0
CD226	rs763361	Blood sugar medication	1.04	(1.02-1.07)	3.00E-04	1	17026	230655	0.0556	+????	NA
		Cluster headaches	1.05	(0.97-1)	5.74E-04	1	11070	217298	0.088	+????	NA
		Dust mite allergy (1)	1.03	(1.01-1.04)	4.58E-04	1	43035	206272	0.0762	+????	NA
		Dust mites allergy (clean) (1)	1.03	(1.01-1.05)	3.75E-04	1	42716	114860	0.0677	+????	NA
		Dust mites allergy (meta) (1)	1.03	(1.01-1.05)	3.96E-04	1	42716	123896	0.0695	+????	NA
		**Hypothyroidism**	**1.05**	**(1.04-1.08)**	**8.11E-11**	**3**	**35428**	**412577**	**5.49E-08**	**++?+?**	0
		Lobular carcinoma in situ	0.72	(0.6-0.86)	3.91E-04	1	246	11855	0.0692	-????	NA
		Multiple sclerosis	1.07	(1.03-1.12)	3.78E-04	3	8113	539081	0.0678	+?+?+	0
		Viral hepatitis	1.38	(1.18-1.61)	6.08E-05	2	323	30516	0.0164	??++?	39.93
TYK2	rs12720356	Acne	1.08	(1.03-1.12)	4.21E-04	2	15020	198330	0.0718	+??-?	0
		Gallstones	1.08	(1.04-1.13)	0.0005	1	13300	120654	0.0804	+????	NA
		T2D	1.07	(1.03-1.12)	4.10E-04	3	16354	386040	0.07078	++?+?	0
		Tonsillectomy	1.06	(1.03-1.09)	5.06E-05	1	58942	109420	0.014	+????	NA
TYK2	rs34536443	Anorexia	6.36	(2.4-16.81)	2.03E-04	1	21	9045	0.0409	???+?	NA
		**Any immune disease**	**0.91**	**(0.88-0.93)**	**4.27E-12**	**1**	**112148**	**173986**	**3.48E-09**	**-????**	NA
		**Hypothyroidism**	**0.88**	**(0.83-0.93)**	**1.19E-06**	**3**	**23145**	**233757**	**0.00045**	**--?+?**	55.86
		Hypoventilation	3.65	(1.76-7.56)	4.86E-04	1	55	10130	0.0789	???+?	NA
		Pituitary hypofunction	2.56	(1.49-4.35)	5.95E-04	1	163	10437	0.09	???+?	NA
		Tonsillectomy	1.09	(1.04-1.12)	8.28E-05	1	58942	109420	0.0205	+????	NA
GPR35	rs4676410	Any allergy (2)	0.97	(0.95-0.98)	6.16E-05	1	66627	106999	0.0164	-????	NA
		Chemotherapy	1.08	(1.04-1.12)	1.47E-04	2	8339	266624	0.0324	+??+?	0
		Essential hypertension	0.9	(0.85-0.95)	4.19E-04	2	4193	30694	0.0718	??--?	74.25
		No allergies (2)	1.03	(1.02-1.04)	5.58E-04	1	115400	124518	0.088	+????	NA
		Pollen allergy (2)	0.96	(0.94-0.98)	6.11E-04	1	39115	110451	0.0917	-????	NA
		Rhinitis (2)	0.96	(0.94-0.98)	1.23E-05	1	53635	117562	0.00388	-????	NA
		Seasonal allergies (broad) (2)	0.96	(0.95-0.98)	1.52E-04	1	50993	109231	0.0327	-????	NA
		Weeds allergy (2)	0.96	(0.94-0.98)	5.73E-04	1	28025	111931	0.088	-????	NA
GPR65	rs3742704	Asthma	1.04	(1.02-1.06)	4.58E-05	4	90712	455177	0.013	+-?++	54.04
		Eczema	1.04	(1.03-1.06)	9.80E-05	2	62003	558968	0.0231	+-???	0
		Obstructive sleep apnea	1.47	(1.17-1.82)	6.83E-04	1	404	10143	0.0993	???+?	NA
		Speech and language disorder	1.51	(1.23-1.85)	6.82E-05	1	480	9421	0.0179	???+?	NA
GUCY1A3	rs13139571	Death: Diabetes (type II or I)	0.65	(0.51-0.83)	2.55E-04	1	248	21044	0.0491	??-??	NA
		High cholesterol medication	0.98	(1.03-1.08)	5.74E-04	1	118184	407440	0.088	-????	NA
IFIH1	rs1990760	AMD	1.1	(1.04-1.15)	6.53E-04	2	3090	87927	0.0964	+?+??	74.87
		**Asthma**	**1.04**	**(1.02-1.05)**	**1.11E-07**	**5**	**57182**	**269499**	**5.24E-05**	**+++++**	**0**
		CAD	0.96	(0.94-0.98)	5.16E-04	3	35199	320665	0.0829	-?+?-	32.97
		Ever pregnant	0.97	(0.94-0.98)	3.10E-05	1	87019	46269	0.00937	-????	NA

		Hypothyroidism	0.96	(0.94-0.98)	1.64E-04	3	23155	234399	0.0342	--?-?	0
		Took blood thinners	0.96	(0.94-0.98)	3.20E-04	1	22985	236431	0.0588	-????	NA
		Wheat allergy (3)	1.11	(1.05-1.17)	2.19E-04	1	2720	115036	0.0434	+????	NA
		Wheat allergy (clean) (3)	1.1	(1.04-1.16)	6.93E-04	1	2684	80097	0.0999	+????	NA
IRF5	rs10488631	Acid-base balance disorder	2.2	(1.41-3.46)	5.80E-04	1	55	12801	0.0885	???+?	NA
		**Hypothyroidism**	**1.08**	**(1.05-1.12)**	**5.78E-07**	**3**	**23182**	**236240**	**2.36E-04**	**++?+?**	8.01
		Systolic/diastolic heart failure	3.69	(2.04-6.69)	1.57E-05	1	25	13191	0.00483	???+?	NA
IRF5	rs2004640	Psoriasis	0.9	(0.85-0.95)	6.59E-05	4	3737	144879	0.0174	?----	0
IRF5	rs3823536	Psoriasis	1.11	(1.06-1.18)	8.92E-05	4	3690	145494	0.0217	?++-+	0
B. Known cardiovascular disease-associated SNPs											
F11	rs4253399	Pulmonary embolism	1.17	(1.07-1.28)	6.80E-04	1	949	111077	0.0993	?+???	NA
GDF15	rs17724992	Acid reflux (4)	0.97	(0.96-0.99)	1.56E-04	2	67776	299571	0.0333	--???	0
		ADHD	0.98	(0.95-0.99)	5.61E-04	1	38253	463544	0.088	-????	NA
		Ankylosing spondylitis	0.86	(0.8-0.93)	6.92E-05	2	2814	113626	0.0179	?-??-	0
		**Blood pressure medication** (5)	**0.97**	**(0.96-0.98)**	**1.76E-07**	**1**	**125406**	**394753**	**7.63E-05**	**-????**	NA
		CAD	0.96	(0.94-0.98)	1.40E-05	2	40900	554363	0.00437	-???-	0
		Crohn's disease	0.93	(0.9-0.96)	4.12E-05	2	11229	522948	0.0119	-???-	0
		**CVD** (5)	**0.97**	**(0.96-0.98)**	**1.40E-06**	**1**	**148577**	**388405**	**5.11E-04**	**-????**	NA
		**GERD** (4)	**0.97**	**(0.96-0.98)**	**6.11E-07**	**1**	**130654**	**384572**	**2.46E-04**	**-????**	NA
		Healthy old	1.05	(1.03-1.07)	1.23E-05	1	25481	314694	0.00388	+????	NA
		Heart attack	0.95	(0.92-0.98)	5.71E-04	2	12948	613899	0.088	--???	0.51
		**Heart metabolic disease** (5)	**0.97**	**(0.97-0.98)**	**3.08E-09**	**1**	**275944**	**209302**	**1.86E-06**	**-????**	NA
		Hemorrhoids	0.98	(0.95-0.99)	5.50E-04	1	60280	104280	0.0872	-????	NA
		**High blood pressure** (5)	**0.97**	**(0.97-0.98)**	**7.64E-09**	**2**	**151511**	**465686**	**4.42E-06**	**--???**	80.26
		Insomnia (6)	0.98	(0.97-0.99)	1.22E-05	1	140208	376764	0.00388	-????	NA
		Low HDL	0.97	(0.95-0.99)	6.16E-04	1	47006	172343	0.0919	-????	NA
		Psoriasis	0.97	(0.95-0.98)	2.59E-04	2	29205	600126	0.0493	--???	0
		Sleep medication (6)	0.99	(0.97-0.99)	4.51E-04	1	109844	365576	0.0759	-????	NA
KNG1	rs5030062	Bleeding tendency	0.88	(0.81-0.94)	4.05E-04	1	1574	85223	0.07	-????	NA
		Blood clots	1.07	(1.03-1.1)	1.57E-04	3	7495	275151	0.0333	+?+-?	59.82
LGALS3	rs2274273	CAD	1.05	(1.02-1.07)	1.48E-04	1	17875	469980	0.0324	+????	NA
		Ever pregnant	1.03	(1.01-1.05)	4.55E-04	1	170448	76051	0.076	+????	NA
		Excess hair	1.04	(1.02-1.06)	2.71E-04	1	28355	95842	0.0505	+????	NA
		High plasma glucose	1.02	(1.01-1.04)	6.70E-04	1	50820	415290	0.0984	+????	NA
		Parkinson's disease (7)	0.94	(0.92-0.97)	1.01E-04	1	8615	608014	0.0235	-????	NA
		Parkinson's disease (all population) (7)	0.94	(0.92-0.97)	1.09E-04	1	8615	623197	0.0249	-????	NA
PNPLA3	rs738409	Aspirin AE: liver	1.57	(1.27-1.93)	5.26E-05	1	204	47611	0.0145	+????	NA
		Gallstones (8)	0.95	(0.93-0.98)	2.70E-04	2	18208	258768	0.0505	--????	0
		Gallstones (broad definition) (8)	0.95	(0.92-0.98)	4.72E-04	1	13300	120654	0.077	-????	NA
		Gout	0.93	(0.88-0.96)	4.05E-05	2	8064	237845	0.0118	--???	67.96
		**High cholesterol** (9)	**0.96**	**(0.94-0.97)**	**1.59E-07**	**2**	**101646**	**180947**	**7.11E-05**	**--???**	0
		High cholesterol medication (9)	0.97	(0.95-0.98)	1.96E-04	1	66819	193997	0.0398	-????	NA
		Ibuprofen AE: liver	1.43	(1.21-1.69)	4.57E-05	1	336	48609	0.013	+????	NA
		**Severe acne**	**0.9**	**(0.88-0.93)**	**1.47E-11**	**1**	**14812**	**187018**	**1.14E-08**	**-????**	NA
SLC30A8	rs13266634	High cholesterol medication	0.98	(0.97-1)	3.42E-04	1	118624	409363	0.0626	-????	NA

		Tachycardia	1.75	(1.29-2.36)	2.66E-04	1	89	12759	0.0502	???+?	NA
C. Known immune disease-associated SNPs											
CDHR3	rs6967330	Psoriatic arthropathy	2.5	(1.53-4.09)	2.59E-04	1	37	11000	0.0493	???+?	NA
D. Known neurodegenerative disease-associated SNPs											
TMEM175	rs34311866	Heart metabolic disease	1.1	(1.04-1.15)	6.95E-04	1	37694	6586	0.0999	+????	NA

*Putative novel associations reaching FDR<0.1 are shown. Associations reaching Bonferroni-corrected significance (P<1.8e-6) are highlighted in bold. Full meta-PheWAS results are provided in Supplementary file 1.
^ Effect of minor allele. Refer to Supplementary Data file 1 for detailed information.
$ Direction of effect in 23andMe, Genomics plc UK Biobank, FINRISK, CHOP and GWAS, respectively.
Numbers in parentheses after the Phenotype name identify related/non-independent phenotypic endpoints

Supplementary Table 6. Replication of potential novel associations in the latest release of UK Biobank

				PheWAS meta-analysis				UK Biobank v2					META-ANALYSIS $	
Gene	SNP	Phenotype*	MAF	*N*	Dir#	OR (CI95)	*P* value	UK Biobank field ID	*N* cases/ *N* controls	Power^	Dir	*P* value	*Z*	*P* value
**A. Strengthened association in meta-analysis with UK Biobank v2; P<0.05 in UK Biobank v2**														
CD226	**rs763361**	**Hypothyroidism**	**0.475**	**3**	**++?+?**	**1.05 (1.04-1.08)**	**8.11e-11**	**20002_1226**	**16376/320783**	**0.991**	**+**	**4.71E-09**	**7.85**	**2.11e-15**
F11	rs4253399	Pulmonary embolism	0.39	1	?+???	1.17 (1.07-1.28)	0.00068	20002_1093	2801/334358	0.999	+	5.42E-13	7.12	5.42e-13
GDF15	**rs17724992**	**High blood pressure (1)**	**0.266**	**2**	**--???**	**0.97 (0.97-0.98)**	**7.64e-9**	**6150_4**	**91033/245650**	**0.999**	**-**	**4.41E-05**	**-7.17**	**3.85e-13**
		**Blood pressure medication (1)**	**0.266**	**1**	**-????**	**0.97 (0.96-0.98)**	**1.76e-7**	**6177_2**	**38548/116154**	**0.904**	**-**	**4.70E-05**	**-6.35**	**1.10e-10**
		**Acid reflux**	**0.266**	**2**	**--???**	**0.97 (0.96-0.99)**	**0.000156**	**20002_1138**	**14316/322843**	**0.614**	**-**	**0.011**	**-4.11**	**1.98e-5**
		Heart attack	0.266	2	--???	0.95 (0.92-0.98)	0.000571	20002_1075	7735/329424	0.803	-	0.00338	-3.56	0.00018
GPR65	rs3742704	Asthma	0.086	4	+-?++	1.04 (1.02-1.06)	4.58e-5	22127	10589/72940	0.328	+	0.0175	4.7	1.29e-6
		Eczema	0.086	2	+-???	1.04 (1.03-1.06)	9.80e-5	20002_1452	8718/328441	0.305	+	0.0259	4.28	9.24e-6
GUCY1A3	rs13139571	High cholesterol medication	0.245	1	-????	0.98 (1.03-1.08)	0.000574	6177_1	35840/118862	0.539	-	5.47E-05	-4.71	1.23e-6
IFIH1	**rs2111485**	Hypothyroidism	0.41	3	--?-?	0.96 (0.94-0.98)	0.000164	20002_1226	16376/320783	0.947	-	3.45E-12	-6.76	6.74e-12
		**Asthma**	**0.41**	**5**	**+++++**	**1.04 (1.02-1.05)**	**1.11e-7**	**20002_1111**	**39049/298110**	**0.999**	**+**	**2.55E-05**	**5.49**	**2.01e-08**
IRF5	**rs10488631**	**Hypothyroidism**	**0.11**	**3**	**++?+?**	**1.08 (1.05-1.12)**	**5.78e-7**	**20002_1226**	**16376/320783**	**0.99**	**+**	**0.00091**	**5.06**	**2.11e-7**
PNPLA3	**rs738409**	Gallstones	0.228	2	--???	0.95 (0.92-0.98)	0.00027	20002_1162	5573/331586	0.624	-	0.00966	-4.17	1.51e-5
		Gout	0.228	2	--???	0.93 (0.88-0.96)	4.05e-5	20002_1466	4807/332352	0.849	-	6.17E-09	-5.77	3.87e-9
		**High cholesterol (2)**	**0.228**	**2**	**--???**	**0.96 (0.94-0.97)**	**1.59e-7**	**20002_1473**	**41296/295863**	**0.996**	**-**	**9.35E-05**	**-5.6**	**1.06e-8**
		High cholesterol medication (2)	0.228	1	-????	0.97 (0.95-0.98)	0.000196	6177_1	35840/118862	0.856	-	0.0232	-4.03	2.75e-5
TYK2	**rs34536443**	**Hypothyroidism**	**0.041**	**3**	**--?+?**	**0.88 (0.83-0.93)**	**1.19e-6**	**20002_1226**	**16376/320783**	**0.995**	**-**	**6.69E-11**	**-6.6**	**2.10e-11**
**B. Strengthened association in meta-analysis with UK Biobank v2; P>0.05 in UK Biobank v2**														
CD226	rs763361	Dust mites allergy	0.475	1	+????	1.03 (1.01-1.04)	0.000458	20002_1668	183/336976	0.059	+	0.552	3.35	0.0004
GDF15	rs17724992	Insomnia	0.266	1	-????	0.98 (0.97-0.99)	1.22e-5	20002_1616	321/336838	0.056	-	0.798	-4.26	1.02e-5
		Psoriasis	0.266	2	--???	0.97 (0.95-0.98)	0.000259	20002_1453	3871/333288	0.221	-	0.088	-3.51	0.00022
GPR35	rs4676410	Pollen allergy	0.2	1	-????	0.96 (0.94-0.98)	0.000611	22126	18934/64595	0.803	-	0.102	-3.37	0.00037
PNPLA3	**rs738409**	**Acne**	**0.228**	**1**	**-????**	**0.9 (0.88-0.93)**	**1.47e-11**	**20002_1548**	**189/336970**	**0.14**	**-**	**0.899**	**-6.75**	**7.25e-12**
SLC30A8	rs13266634	High cholesterol medication	0.3	1	-????	0.98 (0.97-1)	0.000342	6153_1	22705/157498	0.461	-	0.104	-3.61	0.00015
TYK2	rs12720356	T2D	0.088	3	++?+?	1.07 (1.03-1.12)	0.00041	20002_1223	2133/335026	0.243	+	0.0539	3.6	0.00016
TYK2	rs34536443	Pituitary hypofunction	0.041	1	???+?	2.56 (1.49-4.35)	0.000595	20002_1430	118/337041	0.851	+	0.105	3.28	0.00053
**C. Lack of replication in UK Biobank v2**														
CARD9	rs4077515	T2D	0.42	4	--?--	0.96 (0.94-0.98)	7.62e-5	20002_1223	2133/335026	0.263	-	0.562	-3.53	0.0002
		Heart attack	0.42	2	--???	0.94 (0.91-0.97)	0.00023	20002_1075	7735/329424	0.967	-	0.0108	-3.12	0.0009
CD226	rs763361	Viral hepatitis	0.475	2	??++?	1.38 (1.18-1.61)	6.08e-5	20002_1156	680/336479	0.999	-	0.266	1.58	0.0566

		Multiple sclerosis	0.475	3	+?+?+	1.07 (1.03-1.12)	0.000378	20002_1261	1228/335931	0.393	+	0.315	1.31	0.0947
CDHR3	rs6967330	Psoriatic arthropathy	0.2	1	???+?	2.5 (1.53-4.09)	0.000259	20002_1477	650/336509	1	-	0.847	-0.19	0.424
GDF15	rs17724992	Ankylosing spondylitis	0.266	2	?-??-	0.86 (0.8-0.93)	6.92e-5	20002_1313	968/336191	0.841	+	0.27	-1.67	0.047
		Crohn's disease	0.266	2	-???-	0.93 (0.9-0.96)	4.12e-5	20002_1462	1032/336127	0.312	-	0.198	-0.68	0.246
		CVD (1)	0.266	1	-????	0.97 (0.96-0.98)	1.40e-6	20002_1066	1096/336063	0.097	+	0.0387	-4.48	3.66e-6
GPR35	rs4676410	Essential hypertension	0.2	2	??--?	0.9 (0.85-0.95)	0.000419	20002_1072	1537/335622	0.654	+	0.176	-2.26	0.01187
GPR65	rs3742704	Obstructive sleep apnea	0.086	1	???+?	1.47 (1.17-1.82)	0.000683	20002_1123	1064/336095	0.999	-	0.837	0.82	0.207
IFIH1	rs2111485	AMD	0.41	2	+?+??	1.1 (1.04-1.15)	0.000653	20002_1528	189/336970	0.151	-	0.59	2.9	0.00185
IRF5	rs10488631	Systolic/diastolic heart failure	0.11	1	???+?	3.69 (2.04-6.69)	1.57e-5	20002_1076	216/336943	0.999	-	0.424	1.55	0.0603
IRF5	rs2004640	Psoriasis	0.47	4	?----	0.9 (0.85-0.95)	6.59e-5	20002_1453	3871/333288	0.996	-	0.00732	-2.57	0.00495
IRF5	rs3823536	Psoriasis	0.47	4	?++-+	1.11 (1.06-1.18)	8.92e-5	20002_1453	3871/333288	0.996	+	0.00326	2.44	0.00735
KNG1	rs5030062	Blood clots	0.384	3	+?+-?	1.07 (1.03-1.1)	0.000157	20002_1068	66/337093	0.067	-	0.488	3.48	0.00025
		Bleeding tendency	0.384	1	-????	0.88 (0.81-0.94)	0.000405	20002_1445	320/336839	0.355	+	0.983	-2.17	0.0149
LGALS3	rs2274273	Parkinson's disease	0.4	1	-????	0.94 (0.92-0.97)	0.000101	20002_1262	604/336555	0.186	-	0.382	-3.66	0.00012
SLC30A8	rs13266634	Tachycardia	0.3	1	???+?	1.75 (1.29-2.36)	0.000266	20002_1487	168/336991	0.998	+	0.912	3.13	0.00088
TYK2	rs12720356	Gallstones	0.088	1	+????	1.08 (1.04-1.13)	0.0005	20002_1162	5573/331586	0.636	+	0.234	3.29	0.0005

[*]Associations reaching Bonferroni-corrected significance (P<1.8e-6) in the discovery PheWAS meta-analysis are highlighted in bold.

[#]Directions of effect in 23andMe, Gplc UK Biobank, FINRISK, CHOP and GWAS

[^]Statistical power to detect an association at P<0.05 in UK Biobank v2 based on the SNP MAF, the number of cases and controls in UK Biobank v2 and the OR observed in the PheWAS meta-analysis

[$]Weighted Z score meta-analysis using the association results from the 23andMe, FINRISK and CHOP PheWAS cohorts (when available), GWAS (when available) and UK Biobank v2. Weights are calculated based on the number of cases and controls. Details are provided in Methods. The Gplc UK Biobank association results are excluded in this meta-analysis.

Supplementary Table 7. Novel association of the *PNPLA3* SNP rs738409 in the 23andMe cohort, before and after adjusting for elevated liver test

	Unadjusted analysis				Elevated liver test-adjusted analysis*			
Phenotype	OR (CI95) ^	*P* value	*N* cases	*N* controls	OR (CI95) ^	*P* value	*N* cases	*N* controls
Severe acne	0.9 (0.88-0.93)	1.47e-11	14812	187018	0.9 (0.88-0.93)	4.34e-12	14646	185390
T2D	1.09 (1.06-1.12)	1.62e-9	15428	262918	1.07 (1.03-1.1)	3.75e-5	13160	226602
High cholesterol	0.96 (0.94-0.98)	1.50e-5	86968	83599	0.95 (0.94-0.97)	1.79e-6	76389	77211
Anti-cholesterol medication	0.97 (0.95-0.98)	0.0002	66819	193997	0.96 (0.94-0.98)	5.77e-6	58115	166268
Gallstones	0.95 (0.92-0.98)	0.00047	13300	120654	0.93 (0.9-0.96)	3.21e-5	11689	104259
Gout	0.94 (0.9-0.98)	0.0036	6452	127120	0.93 (0.88-0.97)	0.0013	5629	109921

*In the elevated liver test-adjusted analysis, self-report of elevated liver test was included as covariate in the logistic regression, and only participants who answered both liver test questions and questions related to the primary phenotype tested were included.

^Odds ratio of the minor allele, which is associated with elevated alanine aminotransferase (ALT) levels

Supplementary Table 8. Association of the *IFIH1* SNP rs2111485 (in complete LD with the *IFIH1* missense SNP rs1990760) with asthma in 23andMe cohort, before and after adjusting for autoimmune diseases

Analysis	*N* cases	*N* controls	OR (CI95)^	*P* value
unadjusted	28,400	126,511	1.033 (1.014-1.053)	6.5e-4
adjusted*	22,028	95,775	1.034 (1.013-1.057)	1.9e-3

*In the autoimmune disease-adjusted analysis, self-report of autoimmune disorder was included as covariate in the logistic regression, and only participants who answered both autoimmune and asthma-related questions were included.
^Odds ratio of the minor allele, which is known to protect against multiple autoimmune diseases.

Supplementary References

1. Hampe, J. et al. A genome-wide association scan of nonsynonymous SNPs identifies a susceptibility variant for Crohn disease in ATG16L1. *Nat Genet* **39**, 207-211 (2007).
2. Rioux, J.D. et al. Genome-wide association study identifies new susceptibility loci for Crohn disease and implicates autophagy in disease pathogenesis. *Nat Genet.* **39**, 596-604. Epub 2007 Apr 2015. (2007).
3. Jostins, L. et al. Host-microbe interactions have shaped the genetic architecture of inflammatory bowel disease. *Nature* **491**, 119-124 (2012).
4. Lassen, K.G. et al. Atg16L1 T300A variant decreases selective autophagy resulting in altered cytokine signaling and decreased antibacterial defense. *Proc Natl Acad Sci U S A.* **111**, 7741-7746. doi: 7710.1073/pnas.1407001111. Epub 1407002014 May 1407001112. (2014).
5. Murthy, A. et al. A Crohn's disease variant in Atg16l1 enhances its degradation by caspase 3. *Nature.* **506**, 456-462. doi: 410.1038/nature13044. Epub 12014 Feb 13019. (2014).
6. Franke, A. et al. Genome-wide meta-analysis increases to 71 the number of confirmed Crohn's disease susceptibility loci. *Nat Genet.* **42**, 1118-1125. doi: 1110.1038/ng.1717. (2010).
7. Fairfax, B.P. et al. Innate immune activity conditions the effect of regulatory variants upon monocyte gene expression. *Science.* **343**, 1246949. doi: 1246910.1241126/science.1246949. (2014).
8. Janse, M. et al. Three ulcerative colitis susceptibility loci are associated with primary sclerosing cholangitis and indicate a role for IL2, REL, and CARD9. *Hepatology.* **53**, 1977-1985. doi: 1910.1002/hep.24307. Epub 22011 May 24302. (2011).
9. Rivas, M.A. et al. Deep resequencing of GWAS loci identifies independent rare variants associated with inflammatory bowel disease. *Nat Genet.* **43**, 1066-1073. doi: 1010.1038/ng.1952. (2011).
10. Lanternier, F. et al. Deep dermatophytosis and inherited CARD9 deficiency. *N Engl J Med.* **369**, 1704-1714. doi: 1710.1056/NEJMoa1208487. Epub 1202013 Oct 1208416. (2013).
11. Ruland, J. CARD9 signaling in the innate immune response. *Ann N Y Acad Sci.* **1143:35-44.**, 10.1196/annals.1443.1024. (2008).
12. Elishmereni, M., Bachelet, I. & Levi-Schaffer, F. DNAM-1: an amplifier of immune responses as a therapeutic target in various disorders. *Curr Opin Investig Drugs.* **9**, 491-496. (2008).
13. Xiong, P., Sang, H.W. & Zhu, M. Critical roles of co-activation receptor DNAX accessory molecule-1 in natural killer cell immunity. *Immunology.* **146**, 369-378. doi: 310.1111/imm.12516. Epub 12015 Sep 12528. (2015).
14. Onengut-Gumuscu, S. et al. Fine mapping of type 1 diabetes susceptibility loci and evidence for colocalization of causal variants with lymphoid gene enhancers. *Nat Genet.* **47**, 381-386. doi: 310.1038/ng.3245. Epub 2015 Mar 1039. (2015).
15. Gieger, C. et al. New gene functions in megakaryopoiesis and platelet formation. *Nature.* **480**, 201-208. doi: 210.1038/nature10659. (2011).
16. Gross, C.C. et al. Impaired NK-mediated regulation of T-cell activity in multiple sclerosis is reconstituted by IL-2 receptor modulation. *Proc Natl Acad Sci U S A.* **113**, E2973-2982. doi: 2910.1073/pnas.1524924113. Epub 1524922016 May 1524924119. (2016).
17. Lofgren, S.E. et al. A 3'-untranslated region variant is associated with impaired expression of CD226 in T and natural killer T cells and is associated with susceptibility to systemic lupus erythematosus. *Arthritis Rheum.* **62**, 3404-3414. doi: 3410.1002/art.27677. (2010).

18. Divorty, N., Mackenzie, A.E., Nicklin, S.A. & Milligan, G. G protein-coupled receptor 35: an emerging target in inflammatory and cardiovascular disease. *Front Pharmacol.* **6:41.**, 10.3389/fphar.2015.00041. eCollection 02015. (2015).
19. Lassen, K.G. et al. Genetic Coding Variant in GPR65 Alters Lysosomal pH and Links Lysosomal Dysfunction with Colitis Risk. *Immunity.* **44**, 1392-1405. doi: 1310.1016/j.immuni.2016.1305.1007. Epub 2016 Jun 1397. (2016).
20. Looney, B.M., Xia, C.Q., Concannon, P., Ostrov, D.A. & Clare-Salzler, M.J. Effects of type 1 diabetes-associated IFIH1 polymorphisms on MDA5 function and expression. *Curr Diab Rep.* **15**, 96. doi: 10.1007/s11892-11015-10656-11898. (2015).
21. Oda, H. et al. Aicardi-Goutieres syndrome is caused by IFIH1 mutations. *Am J Hum Genet.* **95**, 121-125. doi: 110.1016/j.ajhg.2014.1006.1007. (2014).
22. Rice, G.I. et al. Gain-of-function mutations in IFIH1 cause a spectrum of human disease phenotypes associated with upregulated type I interferon signaling. *Nat Genet.* **46**, 503-509. doi: 510.1038/ng.2933. Epub 2014 Mar 1030. (2014).
23. Rutsch, F. et al. A specific IFIH1 gain-of-function mutation causes Singleton-Merten syndrome. *Am J Hum Genet.* **96**, 275-282. doi: 210.1016/j.ajhg.2014.1012.1014. Epub 2015 Jan 1022. (2015).
24. Asgari, S. et al. Severe viral respiratory infections in children with IFIH1 loss-of-function mutations. *Proc Natl Acad Sci U S A* **17**, 1704259114 (2017).
25. Lamborn, I.T. et al. Recurrent rhinovirus infections in a child with inherited MDA5 deficiency. *J Exp Med.* **214**, 1949-1972. doi: 1910.1084/jem.20161759. Epub 20162017 Jun 20161712. (2017).
26. Budu-Aggrey, A. et al. A rare coding allele in IFIH1 is protective for psoriatic arthritis. *Ann Rheum Dis.* **76**, 1321-1324. doi: 1310.1136/annrheumdis-2016-210592. Epub 212017 May 210513. (2017).
27. Downes, K. et al. Reduced expression of IFIH1 is protective for type 1 diabetes. *PLoS One.* **5(9).** e12646. doi: 12610.11371/journal.pone.0012646. (2010).
28. Jin, Y. et al. Genome-wide association analyses identify 13 new susceptibility loci for generalized vitiligo. *Nat Genet.* **44**, 676-680. doi: 610.1038/ng.2272. (2012).
29. Jin, Y., Andersen, G.H., Santorico, S.A. & Spritz, R.A. Multiple Functional Variants of IFIH1, a Gene Involved in Triggering Innate Immune Responses, Protect against Vitiligo. *J Invest Dermatol.* **137**, 522-524. doi: 510.1016/j.jid.2016.1009.1021. Epub 2016 Oct 1015. (2017).
30. Li, Y. et al. Carriers of rare missense variants in IFIH1 are protected from psoriasis. *J Invest Dermatol.* **130**, 2768-2772. doi: 2710.1038/jid.2010.2214. Epub 2010 Jul 2729. (2010).
31. Nejentsev, S., Walker, N., Riches, D., Egholm, M. & Todd, J.A. Rare variants of IFIH1, a gene implicated in antiviral responses, protect against type 1 diabetes. *Science.* **324**, 387-389. doi: 310.1126/science.1167728. Epub 1162009 Mar 1167725. (2009).
32. Shigemoto, T. et al. Identification of loss of function mutations in human genes encoding RIG-I and MDA5: implications for resistance to type I diabetes. *J Biol Chem.* **284**, 13348-13354. doi: 13310.11074/jbc.M809449200. Epub 809442009 Mar 809449226. (2009).
33. Smyth, D.J. et al. A genome-wide association study of nonsynonymous SNPs identifies a type 1 diabetes locus in the interferon-induced helicase (IFIH1) region. *Nat Genet.* **38**, 617-619. Epub 2006 May 2014. (2006).
34. Zurawek, M. et al. Cumulative effect of IFIH1 variants and increased gene expression associated with type 1 diabetes. *Diabetes Res Clin Pract.* **107**, 259-266. doi: 210.1016/j.diabres.2014.1011.1008. Epub 2014 Dec 1014. (2015).

35. Bentham, J. et al. Genetic association analyses implicate aberrant regulation of innate and adaptive immunity genes in the pathogenesis of systemic lupus erythematosus. *Nat Genet* **47**, 1457-1464 (2015).
36. Liu, J.Z. et al. Association analyses identify 38 susceptibility loci for inflammatory bowel disease and highlight shared genetic risk across populations. *Nat Genet.* **47**, 979-986. doi: 910.1038/ng.3359. Epub 2015 Jul 1020. (2015).
37. Gorman, J.A. et al. The A946T variant of the RNA sensor IFIH1 mediates an interferon program that limits viral infection but increases the risk for autoimmunity. *Nat Immunol.* **18**, 744-752. doi: 710.1038/ni.3766. Epub 2017 May 1029. (2017).
38. Okada, Y. et al. Genetics of rheumatoid arthritis contributes to biology and drug discovery. *Nature* **506**, 376-381 (2014).
39. Taylor, K.E. et al. Genome-Wide Association Analysis Reveals Genetic Heterogeneity of Sjogren's Syndrome According to Ancestry. *Arthritis Rheumatol.* **69**, 1294-1305. doi: 1210.1002/art.40040. Epub 42017 May 40049. (2017).
40. Liu, X. et al. Genome-wide meta-analyses identify three loci associated with primary biliary cirrhosis. *Nat Genet.* **42**, 658-660. doi: 610.1038/ng.1627. Epub 2010 Jul 1018. (2010).
41. Radstake, T.R. et al. Genome-wide association study of systemic sclerosis identifies CD247 as a new susceptibility locus. *Nat Genet.* **42**, 426-429. doi: 410.1038/ng.1565. Epub 2010 Apr 1011. (2010).
42. Hedl, M. & Abraham, C. IRF5 risk polymorphisms contribute to interindividual variance in pattern recognition receptor-mediated cytokine secretion in human monocyte-derived cells. *J Immunol.* **188**, 5348-5356. doi: 5310.4049/jimmunol.1103319. Epub 1102012 Apr 1103327. (2012).
43. Hedl, M., Yan, J. & Abraham, C. IRF5 and IRF5 Disease-Risk Variants Increase Glycolysis and Human M1 Macrophage Polarization by Regulating Proximal Signaling and Akt2 Activation. *Cell Rep.* **16**, 2442-2455. doi: 2410.1016/j.celrep.2016.2407.2060. Epub 2016 Aug 2418. (2016).
44. Niewold, T.B. et al. IRF5 haplotypes demonstrate diverse serological associations which predict serum interferon alpha activity and explain the majority of the genetic association with systemic lupus erythematosus. *Ann Rheum Dis.* **71**, 463-468. doi: 410.1136/annrheumdis-2011-200463. Epub 202011 Nov 200416. (2012).
45. Kreins, A.Y. et al. Human TYK2 deficiency: Mycobacterial and viral infections without hyper-IgE syndrome. *J Exp Med.* **212**, 1641-1662. doi: 1610.1084/jem.20140280. Epub 20142015 Aug 20140224. (2015).
46. Minegishi, Y. et al. Human tyrosine kinase 2 deficiency reveals its requisite roles in multiple cytokine signals involved in innate and acquired immunity. *Immunity.* **25**, 745-755. (2006).
47. Tsoi, L.C. et al. Identification of 15 new psoriasis susceptibility loci highlights the role of innate immunity. *Nat Genet.* **44**, 1341-1348. doi: 1310.1038/ng.2467. Epub 2012 Nov 1311. (2012).
48. Beecham, A.H. et al. Analysis of immune-related loci identifies 48 new susceptibility variants for multiple sclerosis. *Nat Genet.* **45**, 1353-1360. doi: 1310.1038/ng.2770. Epub 2013 Sep 1329. (2013).
49. Diogo, D. et al. TYK2 protein-coding variants protect against rheumatoid arthritis and autoimmunity, with no evidence of major pleiotropic effects on non-autoimmune complex traits. *PLoS One.* **10**, e0122271. doi: 0122210.0121371/journal.pone.0122271. eCollection 0122015. (2015).

50. Couturier, N. et al. Tyrosine kinase 2 variant influences T lymphocyte polarization and multiple sclerosis susceptibility. *Brain.* **134**, 693-703. doi: 610.1093/brain/awr1010. (2011).
51. Dendrou, C.A. et al. Resolving TYK2 locus genotype-to-phenotype differences in autoimmunity. *Sci Transl Med.* **8**, 363ra149. (2016).
52. Bonnelykke, K. et al. A genome-wide association study identifies CDHR3 as a susceptibility locus for early childhood asthma with severe exacerbations. *Nat Genet.* **46**, 51-55. doi: 10.1038/ng.2830. Epub 2013 Nov 1017. (2014).
53. Bochkov, Y.A. et al. Cadherin-related family member 3, a childhood asthma susceptibility gene product, mediates rhinovirus C binding and replication. *Proc Natl Acad Sci U S A.* **112**, 5485-5490. doi: 5410.1073/pnas.1421178112. Epub 1421172015 Apr 1421178116. (2015).
54. Asakai, R., Chung, D.W., Ratnoff, O.D. & Davie, E.W. Factor XI (plasma thromboplastin antecedent) deficiency in Ashkenazi Jews is a bleeding disorder that can result from three types of point mutations. *Proc Natl Acad Sci U S A.* **86**, 7667-7671. (1989).
55. Sabater-Lleal, M. et al. A genome-wide association study identifies KNG1 as a genetic determinant of plasma factor XI Level and activated partial thromboplastin time. *Arterioscler Thromb Vasc Biol.* **32**, 2008-2016. doi: 2010.1161/ATVBAHA.2112.248492. Epub 242012 Jun 248414. (2012).
56. Tang, W. et al. Genetic associations for activated partial thromboplastin time and prothrombin time, their gene expression profiles, and risk of coronary artery disease. *Am J Hum Genet.* **91**, 152-162. doi: 110.1016/j.ajhg.2012.1005.1009. Epub 2012 Jun 1014. (2012).
57. Tang, W. et al. A genome-wide association study for venous thromboembolism: the extended cohorts for heart and aging research in genomic epidemiology (CHARGE) consortium. *Genet Epidemiol.* **37**, 512-521. doi: 510.1002/gepi.21731. Epub 22013 May 21735. (2013).
58. Buller, H.R. et al. Factor XI antisense oligonucleotide for prevention of venous thrombosis. *N Engl J Med.* **372**, 232-240. doi: 210.1056/NEJMoa1405760. Epub 1402014 Dec 1405767. (2015).
59. Egeberg, O. Factor XII defect and hemorrhage. Evidence for a new type of hereditary hemostatic disorder. *Thromb Diath Haemorrh.* **23**, 432-440. (1970).
60. Ratnoff, O.D. & Steinberg, A.G. Further studies on the inheritance of Hageman trait. *J Lab Clin Med.* **59**, 980-985. (1962).
61. Dewald, G. & Bork, K. Missense mutations in the coagulation factor XII (Hageman factor) gene in hereditary angioedema with normal C1 inhibitor. *Biochem Biophys Res Commun.* **343**, 1286-1289. doi: 1210.1016/j.bbrc.2006.1203.1092 (2006).
62. Renne, T. et al. Defective thrombus formation in mice lacking coagulation factor XII. *J Exp Med.* **202**, 271-281. Epub 2005 Jul 2011. (2005).
63. Calafell, F. et al. Sequence variation and genetic evolution at the human F12 locus: mapping quantitative trait nucleotides that influence FXII plasma levels. *Hum Mol Genet.* **19**, 517-525. doi: 510.1093/hmg/ddp1517. Epub 2009 Nov 1023. (2010).
64. Houlihan, L.M. et al. Common variants of large effect in F12, KNG1, and HRG are associated with activated partial thromboplastin time. *Am J Hum Genet.* **86**, 626-631. doi: 610.1016/j.ajhg.2010.1002.1016. Epub 2010 Mar 1018. (2010).
65. Locke, A.E. et al. Genetic studies of body mass index yield new insights for obesity biology. *Nature* **518**, 197-206 (2015).
66. Dobrin, R. et al. Dissecting cis regulation of gene expression in human metabolic tissues. *PLoS One* **6**, e23480. doi: 23410.21371/journal.pone.0023480. Epub 0022011 Aug 0023431. (2011).

67. Hong, J.H. et al. GDF15 Is a Novel Biomarker for Impaired Fasting Glucose. *Diabetes Metab J.* **38**, 472-479. doi: 410.4093/dmj.2014.4038.4096.4472. Epub 2014 Dec 4015. (2014).
68. Ehret, G.B. et al. Genetic variants in novel pathways influence blood pressure and cardiovascular disease risk. *Nature.* **478**, 103-109. doi: 110.1038/nature10405. (2011).
69. Rippe, C. et al. Hypertension reduces soluble guanylyl cyclase expression in the mouse aorta via the Notch signaling pathway. *Sci Rep.* **7**, 1334. doi: 1310.1038/s41598-41017-01392-41591. (2017).
70. Herve, D. et al. Loss of alpha1beta1 soluble guanylate cyclase, the major nitric oxide receptor, leads to moyamoya and achalasia. *Am J Hum Genet.* **94**, 385-394. doi: 310.1016/j.ajhg.2014.1001.1018. Epub 2014 Feb 1027. (2014).
71. de Boer, R.A. et al. A genome-wide association study of circulating galectin-3. *PLoS One* **7**, e47385. doi: 47310.41371/journal.pone.0047385. Epub 0042012 Oct 0047389. (2012).
72. Chambers, J.C. et al. Genome-wide association study identifies loci influencing concentrations of liver enzymes in plasma. *Nat Genet.* **43**, 1131-1138. doi: 1110.1038/ng.1970. (2011).
73. Speliotes, E.K., Butler, J.L., Palmer, C.D., Voight, B.F. & Hirschhorn, J.N. PNPLA3 variants specifically confer increased risk for histologic nonalcoholic fatty liver disease but not metabolic disease. *Hepatology.* **52**, 904-912. doi: 910.1002/hep.23768. (2010).
74. Tian, C., Stokowski, R.P., Kershenobich, D., Ballinger, D.G. & Hinds, D.A. Variant in PNPLA3 is associated with alcoholic liver disease. *Nat Genet.* **42**, 21-23. doi: 10.1038/ng.1488. Epub 2009 Nov 1029. (2010).
75. Li, J.Z. et al. Chronic overexpression of PNPLA3I148M in mouse liver causes hepatic steatosis. *J Clin Invest.* **122**, 4130-4144. (2012).
76. Smagris, E. et al. Pnpla3I148M knockin mice accumulate PNPLA3 on lipid droplets and develop hepatic steatosis. *Hepatology.* **61**, 108-118. doi: 110.1002/hep.27242. Epub 22014 Oct 27241. (2015).
77. Sladek, R. et al. A genome-wide association study identifies novel risk loci for type 2 diabetes. *Nature.* **445**, 881-885. Epub 2007 Feb 2011. (2007).
78. Flannick, J. et al. Loss-of-function mutations in SLC30A8 protect against type 2 diabetes. *Nat Genet.* **46**, 357-363. doi: 310.1038/ng.2915. Epub 2014 Mar 1032. (2014).
79. Paisan-Ruiz, C., Lewis, P.A. & Singleton, A.B. LRRK2: cause, risk, and mechanism. *J Parkinsons Dis* **3**, 85-103. doi: 110.3233/JPD-130192. (2013).
80. Nalls, M.A. et al. Large-scale meta-analysis of genome-wide association data identifies six new risk loci for Parkinson's disease. *Nat Genet* **46**, 989-993 (2014).
81. Cang, C., Aranda, K., Seo, Y.J., Gasnier, B. & Ren, D. TMEM175 Is an Organelle K(+) Channel Regulating Lysosomal Function. *Cell.* **162**, 1101-1112. doi: 1110.1016/j.cell.2015.1108.1002. (2015).
82. Jinn, S. et al. TMEM175 deficiency impairs lysosomal and mitochondrial function and increases alpha-synuclein aggregation. *Proc Natl Acad Sci U S A.* **114**, 2389-2394. doi: 2310.1073/pnas.1616332114. Epub 1616332017 Feb 1616332113. (2017).
83. Nikpay, M. et al. A comprehensive 1,000 Genomes-based genome-wide association meta-analysis of coronary artery disease. *Nat Genet.* **47**, 1121-1130. doi: 1110.1038/ng.3396. Epub 2015 Sep 1127. (2015).
84. Ferreira, R.C. et al. Association of IFIH1 and other autoimmunity risk alleles with selective IgA deficiency. *Nat Genet.* **42**, 777-780. doi: 710.1038/ng.1644. Epub 2010 Aug 1038. (2010).

85. Jin, Y. et al. Genome-wide association studies of autoimmune vitiligo identify 23 new risk loci and highlight key pathways and regulatory variants. *Nat Genet.* **48**, 1418-1424. doi: 1410.1038/ng.3680. Epub 2016 Oct 1410. (2016).
86. Buch, S. et al. A genome-wide association study confirms PNPLA3 and identifies TM6SF2 and MBOAT7 as risk loci for alcohol-related cirrhosis. *Nat Genet.* **47**, 1443-1448. doi: 1410.1038/ng.3417. Epub 2015 Oct 1419. (2015).
87. Liu, C.T. et al. Trans-ethnic Meta-analysis and Functional Annotation Illuminates the Genetic Architecture of Fasting Glucose and Insulin. *Am J Hum Genet.* **99**, 56-75. doi: 10.1016/j.ajhg.2016.1005.1006. Epub 2016 Jun 1016. (2016).
88. Mahajan, A. et al. Genome-wide trans-ancestry meta-analysis provides insight into the genetic architecture of type 2 diabetes susceptibility. *Nat Genet.* **46**, 234-244. doi: 210.1038/ng.2897. Epub 2014 Feb 1039. (2014).
