# Supplementary material for: Phenome-wide association studies across large population cohorts support drug target validation
Source: Nat Commun. 2018 Oct 16;9:4285. doi: 10.1038/s41467-018-06540-3 (PMC6191429; doi:10.1038/s41467-018-06540-3)
Supplement: Supplementary file 3 — Description of Additional Supplementary Files [file 41467_2018_6540_MOESM3_ESM.pdf]

Description of Additional Supplementary Files

File Name: Supplementary Data 1
Description: Full summary statistics results from the meta-analysis of PheWAS results in four disease-agnostic cohorts with published GWAS results.
